# A geospatial analysis of local intermediate snail host distributions provides insight into schistosomiasis risk within under-sampled areas of southern Lake Malawi

**DOI:** 10.1186/s13071-024-06353-y

**Published:** 2024-06-27

**Authors:** Amber L. Reed, Mohammad H. Al-Harbi, Peter Makaula, Charlotte Condemine, Josie Hesketh, John Archer, Sam Jones, Sekeleghe A. Kayuni, Janelisa Musaya, Michelle C. Stanton, J. Russell Stothard, Claudio Fronterre, Christopher Jewell

**Affiliations:** 1 Lancaster Medical School, Lancaster University, Bailrigg House, Bailrigg, Lancaster, LA1 4YE UK; 2 Tropical Disease Biology, Liverpool School of Tropical Medicine, Pembroke Place, Liverpool, L3 5QA UK; 3 Ministry of Health, 52367 Buraydah, Saudi Arabia; 4 Malawi Liverpool Wellcome Trust Programme of Clinical Tropical Research, Queen Elizabeth Central Hospital, College of Medicine, P. O. Box 30096, Blantyre, Malawi; 5 Vector Biology, Liverpool School of Tropical Medicine, Pembroke Place, Liverpool, L3 5QA UK; 6 Mathematics and Statistics, Lancaster University, Bailrigg House, Bailrigg, Lancaster, LA1 4YE UK

**Keywords:** *Bulinus*, *Biomphalaria*, Snail abundance, Bayesian multilevel models, Geospatial analysis, Gaussian latent process, Remote sensing

## Abstract

**Background:**

Along the southern shoreline of Lake Malawi, the incidence of schistosomiasis is increasing with snails of the genera *Bulinus* and *Biomphalaria* transmitting urogenital and intestinal schistosomiasis, respectively. Since the underlying distribution of snails is partially known, often being focal, developing pragmatic spatial models that interpolate snail information across under-sampled regions is required to understand and assess current and future risk of schistosomiasis.

**Methods:**

A secondary geospatial analysis of recently collected malacological and environmental survey data was undertaken. Using a Bayesian Poisson latent Gaussian process model, abundance data were fitted for *Bulinus* and *Biomphalaria*. Interpolating the abundance of snails along the shoreline (given their relative distance along the shoreline) was achieved by smoothing, using extracted environmental rainfall, land surface temperature (LST), evapotranspiration, normalised difference vegetation index (NDVI) and soil type covariate data for all predicted locations. Our adopted model used a combination of two-dimensional (2D) and one dimensional (1D) mapping.

**Results:**

A significant association between normalised difference vegetation index (NDVI) and abundance of *Bulinus* spp. was detected (log risk ratio − 0.83, 95% CrI − 1.57, − 0.09). A qualitatively similar association was found between NDVI and *Biomphalaria* sp. but was not statistically significant (log risk ratio − 1.42, 95% CrI − 3.09, 0.10). Analyses of all other environmental data were considered non-significant.

**Conclusions:**

The spatial range in which interpolation of snail distributions is possible appears < 10km owing to fine-scale biotic and abiotic heterogeneities. The forthcoming challenge is to refine geospatial sampling frameworks with future opportunities to map schistosomiasis within actual or predicted snail distributions. In so doing, this would better reveal local environmental transmission possibilities.

**Graphical Abstract:**

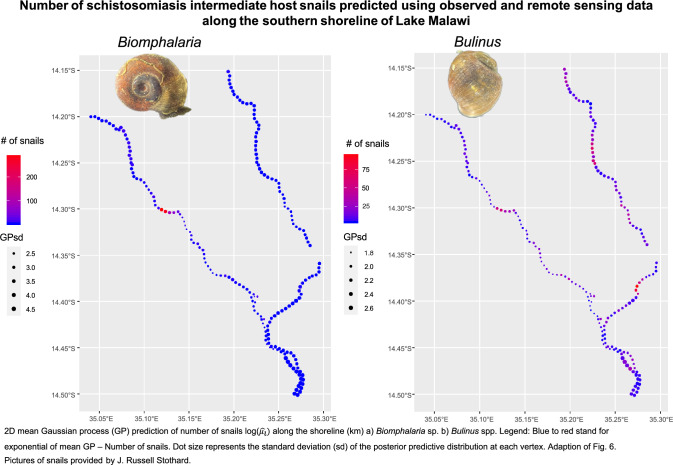

**Supplementary Information:**

The online version contains supplementary material available at 10.1186/s13071-024-06353-y.

## Background

Schistosomiasis is a freshwater snail-borne neglected tropical disease (NTD) common across much of sub-Saharan Africa. Two forms of schistosomiasis occur, urogenital and intestinal schistosomiasis. Their respective transmission can only occur if permissive intermediate snail hosts of the genus *Bulinus* and *Biomphalaria* occur. While various species of *Bulinus* are present in Lake Malawi, with *Bulinus globosus* and *B. nyassanus* responsible for *Schistosoma haematobium* transmission, only in 2017 was *Biomphalaria* first formally noted along its southern shoreline. The expanding distribution of *Biomphalaria pfeifferi* in this area has facilitated transmission of *Schistosoma mansoni*, which causes intestinal schistosomiasis, which has now transitioned from emergence to outbreak [1–3].

Owing to the singular importance of this newly invasive *Bi. pfeifferi*, subsequent malacological surveys were undertaken to track its presence alongside concurrent parasitological surveys in local children in attempt to define the extent of schistosomiasis, particularly intestinal schistosomiasis (IS). These surveys demonstrated the need for further surveillance of freshwater snails, alongside emphasis upon updated and tailored interventions and policies for control of schistosomiasis in this lacustrine setting [1–3]. However, as snail distributions can be patchy or focal, owing to their dependency on local habitats, many gaps in current cartography and predictive mapping are exposed [4]. Indeed, variation in such local characteristics creates difficulties in outlining either permissive or refractory areas where snails may or may not be found, thereby confounding control strategies.

A combination of climate change and human behaviour is thought to be the primary reason for *Biomphalaria* invasion and colonisation into new areas [5]. Characteristics such as vegetation, temperature, rainfall (precipitation), evapotranspiration and soil type have been reported as possible effects on determining snails’ presence and abundance, increasing potential heterogeneity in snail populations over a wide area [5–7]. Changes in climate and seasonal patterns are therefore likely to alter transmission of schistosomiasis over both space and time, increasing the need for identification of snail habitats to target appropriate control interventions [1]. However, although snail distribution within a geographical area can be measured through malacological surveillance, physically collecting freshwater snails is expensive and time consuming, and it is therefore unfeasible to sample every possible location. Thus, effective sampling remains incomplete.

Lake Malawi dominates the eastern side of Malawi, being 600km long and 75km wide. It is known as the second deepest lake in Africa [8] and is vital for those using it for irrigation, agriculture, water supply, fishing industries and tourism [9]. Due to the lack of adequate sanitation in Malawi, human urine and faecal materials continuously contaminate the shoreline facilitating the transmission of schistosomiasis, amongst other water-borne pathogens [10]. In Mangochi District, representing the southern part of Lake Malawi, the eastern side of the lake is mountainous with high elevation (1000–1500m), whereas the western side is flat and with lower elevation (< 500m) [11, 12]. Lower temperatures and higher winds are reported on the eastern side [13], with low-lying areas such as the upper Shire River margins vulnerable to flooding [14]. More broadly, the climate of this southern part of the shoreline is affected by the migration of the Inter-Tropical Converge Zone (ITCZ). This leads to the dry season with cooler temperatures occurring between May and August, hotter temperatures between September to November and wet season between December and April [15, 16]. Rainfall is dependent on altitude and time of the year [17]. Lake water levels vary over time and are at their highest during wet season, which also affects evapotranspiration and outflows to the Shire River [2, 14]. Most important perhaps is an increasing human and livestock population which is leading to more frequent water contact, enhancing opportunities for transmission of schistosomiasis [2, 4].

The World Health Organisation (WHO) has supplied new guidelines to target elimination of schistosomiasis by reducing freshwater snail abundance, thus interrupting transmission [18]. Identifying locations where freshwater snails are most abundant therefore aids targeted control methods, preventing initial infection and re-infection and hence helping eliminate or reduce transmission [19–21].

Here, we undertook a secondary analysis of primary malacological data first reported by Al-Harbi et al. [1] and Kayuni et al. [2]. Our study models the snail distributions as a function of environmental and climate data measured along the shoreline aiming to (i) interpolate and predict the distribution of the snails along the shoreline of Lake Malawi where the snails had not been sampled and (ii) assess the association between environment data and snail distributions. In turn, we hoped to clarify the extent of environmental heterogeneities for schistosomiasis transmission along the shoreline of Lake Malawi and inform the targeting of control programmes to the most appropriate snail breeding sites.

## Methods

The data used in this study consist of observations of snail abundance at a small number of discrete locations on the Lake Malawi shoreline together with remote-sensing data used to describe snail habitat. These are described separately below.

### Snail abundance

**Fig. 1:**
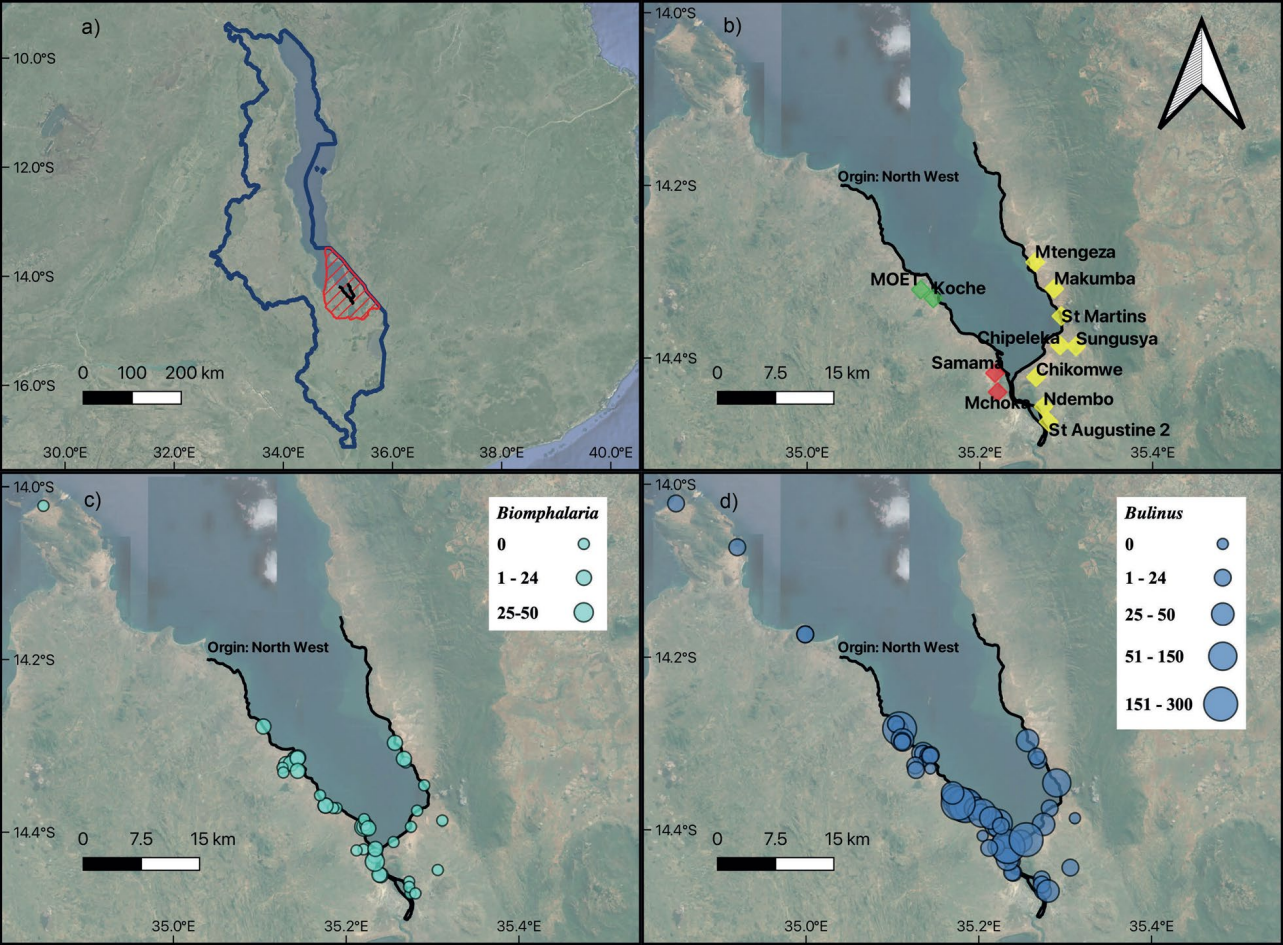
Primary dataset collected data. **a** Map of Malawi in dark blue. Red crossed: study area; black line: prediction points. Parasitological surveys: **b** Primary school locations along the shoreline. Malacological surveys: **c** observed *Biomphalaria* sp. snails; **d** observed *Bulinus* spp. snails

The primary dataset reported in Al-Harbi et al. [1] and Kayuni et al. [2], which this secondary analysis is based on, originally collected malacological surveys between 2017 and 2019 as shown in Fig. 1 and available at Additional file 1: Dataset. Pilot surveillance data from November 2017 identified *Biomphalaria* sp. and *Bulinus* spp. along the shoreline. May/June 2018 and 2019 malacological surveys resampled some of the original locations and added new sites based on satellite imagery or randomly based on their surrounding environment suitable for breeding sites to confirm the emergence and outbreak of IS. The Danish Bilharziasis Laboratory key was used to identify *Bulinus* and *Biomphalaria* according to shell morphology. Figure 1b shows a map of sampling sites, together with their relationship to primary schools in the region, demonstrating the importance of human proximity to the lake shore and hence potential for exposure to infected snails. The snail abundance counts taken from the primary dataset snail counts were numerical counts or in some cases reported as approximate values, e.g. 300 +. In our study we took these approximate values and assumed these values to be the closest lowest value, e.g. 300. The recorded sites considered in our study are shown in Fig. 1c and d.

### Remote sensing data

**Fig. 2:**
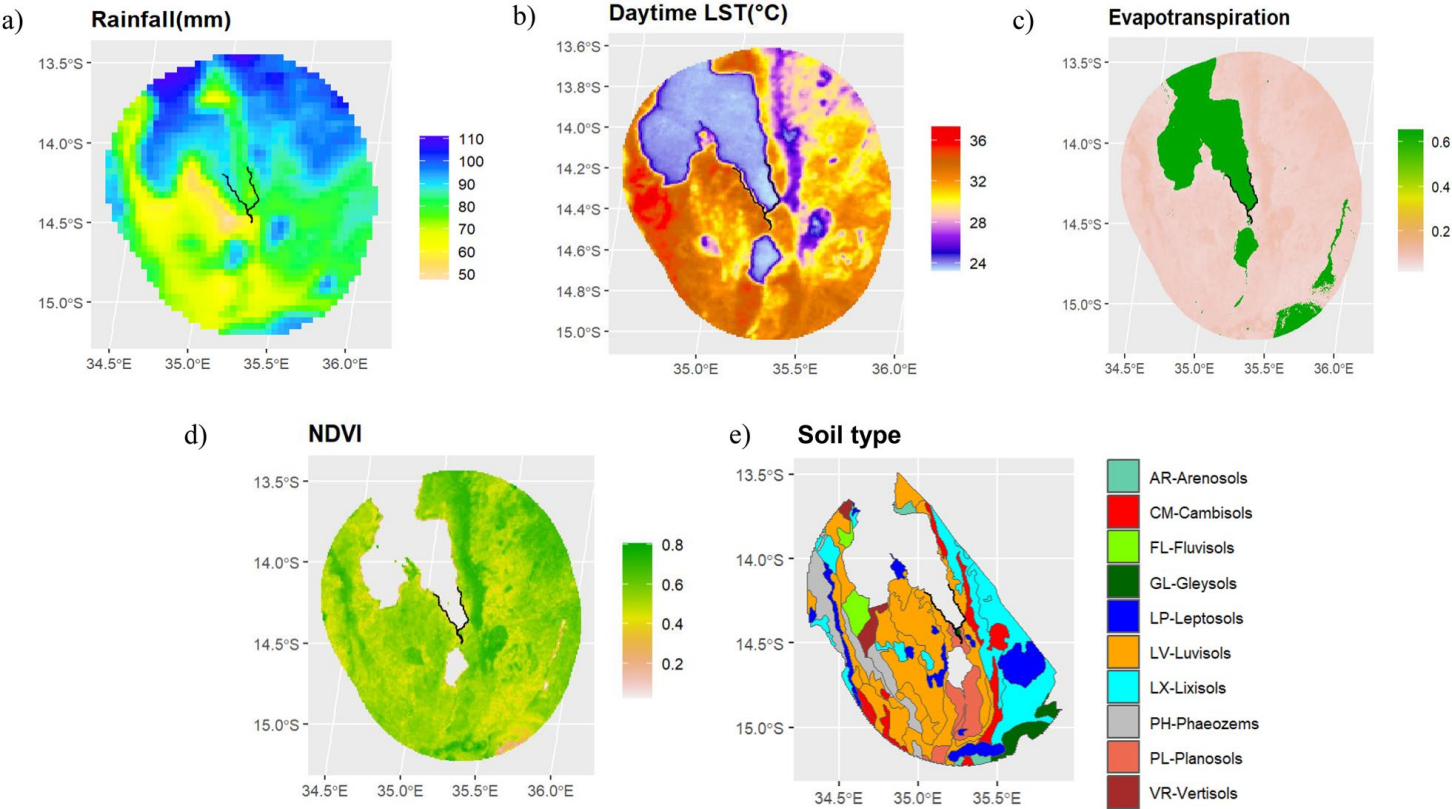
Raster plot of extracted covariate data. **a** Rainfall (mm), **b** daytime land surface temperature (LST) (C), **c** evapotranspiration, **d** normalized difference vegetation index (NDVI). **e** Soil types for southern part of Lake Malawi shoreline, adapted from Dijkshoorn et al. [29]. Black line shows the shoreline template from which covariate values were extracted

Publicly available continuously collected satellite sensory systems were used to extract environmental and climatic data measured adjacent to the shoreline as shown in Fig. 2. Rainfall (millimetres, mm) estimates were extracted between 1 November 2017 and 30 June 2019 from Tropical Applications of Meteorology using SATellite data and ground-based observations (TAMSAT) with a monthly frequency at 4-km resolution [22–24]. Land surface temperature (LST) (°C), evapotranspiration and Normalised Difference Vegetation Index (NDVI) raster data were obtained from Land Processes Distribution Active Archive Center (LPDAAC) [25–28]. LST data between 1 November 2017 to 30 June 2019 were extracted from Moderate Resolution Imaging Spectroradiometer (MODIS)/Terra LST/Emissivity 8-Day L3 Global 1 km SIN Grid raster (MOD11A2v061) [25]. Evapotranspiration data were extracted between 1 January 2014 to 1 January 2019 (5-year time frame) from Modis/Terra Evapotranspiration Gap-Filled Yearly L4 Global 500 m SIN Grid raster (MOD16A3GFv061) [26]. NDVI data between 1 November 2017 to 30 June 2019 were extracted from Modis/Terra vegetation indices 16-Day L3 Global 1 km SIN Grid raster (MOD13A2v0061) [27]. Soil type polygon data were taken from the International Soil Reference and Information Centre (ISRIC) World Soil Information and were derived from the Soil Terrain Database for Malawi (SOTER) at a scale of 1:1 m [29]. After extracting the values, the temporal covariates were aggregated by taking the mean of the values over the time frame.

### Construction of 200 prediction points along the shoreline

Snail abundance was predicted in 1D representation to allow us to interpolate the values along the whole linestring. We made this assumption on the basis that snails live along the shoreline, in habitats that are associated with human water contact and entry, so correlations between snail locations are affected by distance along the shoreline and not, for example, by stretches of deep, open water, e.g. mouth of a bay.

The 1D shoreline was represented by computing the distances between a sequence of 200 vertices obtained from the 2D linestring representation. To achieve this, we used the following method: (i) a 2D linestring was drawn by hand following the shoreline as shown by Google Satellite imagery (Fig. 1); (ii) the linestring was re-sampled to 4000 equally spaced vertices; (iii) each observed sampling site location was snapped to its nearest vertex; (iv) the distance along the line from the origin (northwest-most vertex) to each of the snapper observed sampling site locations was computed (Additional file 2 Fig. S1 and Fig. S2). Additionally, we sub-sampled the 4000 vertices at equal intervals to a set of 200 prediction points.

### Extraction of remote sensing data to linestring vertices

The covariate data were created by extracting the values of each remotely sensed covariate layer data variable surface at each of 200 linestring vertices. To do this, the mean of raster pixels within a 1-km buffer around each vertex was computed. Where missing values were found for a vertex, the buffer took the calculated mean value for the previous corresponding vertex working away from the origin. In cases where missing values were present as the first sampling point, the next collected value was taken.

### One-dimensional Poisson latent Gaussian process regression

#### Bayesian multilevel model

A Bayesian Poisson multilevel model (BMLM) with a Gaussian latent process (GP) was developed using STAN programming language, which uses a Markov Chain Monte Carlo (MCMC) algorithm to regress snail abundance data onto the remotely sensed covariate data, accounting for (1D) spatial correlation along the shoreline. We assumed that the number of snails observed at a sampling location was Poisson distributed, with log-mean given by a coefficient-weighted sum of the covariates plus a spatially correlated error term. Covariance between the error terms was represented at the sum of spatially correlated variance (using quadratic, exponential, or Matérn) *κ* uncorrelated (or nugget) variance [30]. Suitably weakly informative priors were applied to the model coefficients and variance terms, with MCMC run for 10,000 iterations. Posterior summaries (mean and 95% credibility intervals (Crl)) were computed for the fitted model as well as predictive distributions for each of the linestring vertices conditional on the data. All data processing and analysis were performed in R version 4.1.1. See supplementary information (Additional file 3: Model Formulation) for a mathematical explanation of the model.

## Results

### Observed data

**Fig. 3:**
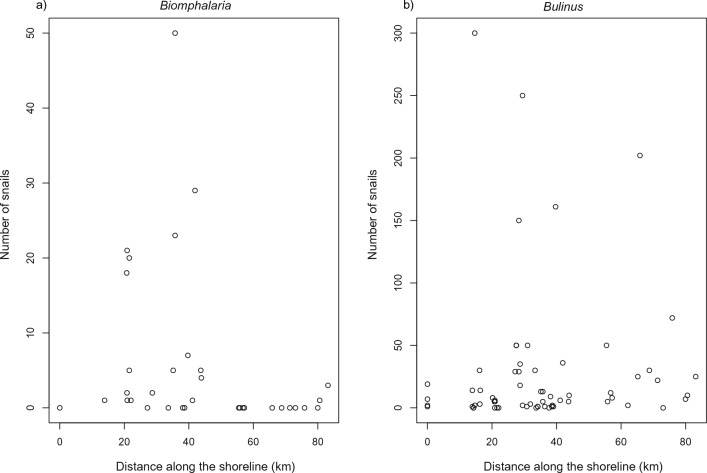
Scatter plot of absolute snail numbers observed at sampling points versus distance along the shoreline in km. **a** *Biomphalaria* sp.; **b** *Bulinus *spp

After cross-checking the observation data, as shown in Fig. 3, we obtained 33 locations where *Biomphalaria* sp. and 63 locations where *Bulinus* spp. were present. The mean number of snails for *Biomphalaria* sp. was 6.03, ranging from 0 to 50 snails, with the most snails found at 46.17km along the shoreline from the origin. The mean number of snails for *Bulinus *spp. was 28.20, ranging from 0 to 300 snails, with the most snails found at 14.66km along the shoreline from the origin. For observed *Biomphalaria* sp. data, the extracted environmental data ranges were rainfall with mean 78.8 (63.01–89.51)mm and LST with mean 29.68 (24.97–32.44) °C. For *Bulinus* spp. data, the extracted environmental data ranges were rainfall with mean 80.62 (63.01–89.5) mm and LST with mean 30.28 (24.97–32.44) °C). Additional file 3 shows the observed data for 1D (Additional file 4: Fig. S1) and 2D (Additional file 4: Fig. S2 and Fig. S3). A histogram of the centred and scaled covariates is shown in Additional file 5: Fig. S1.

### Environmental data prediction points

**Fig. 4:**
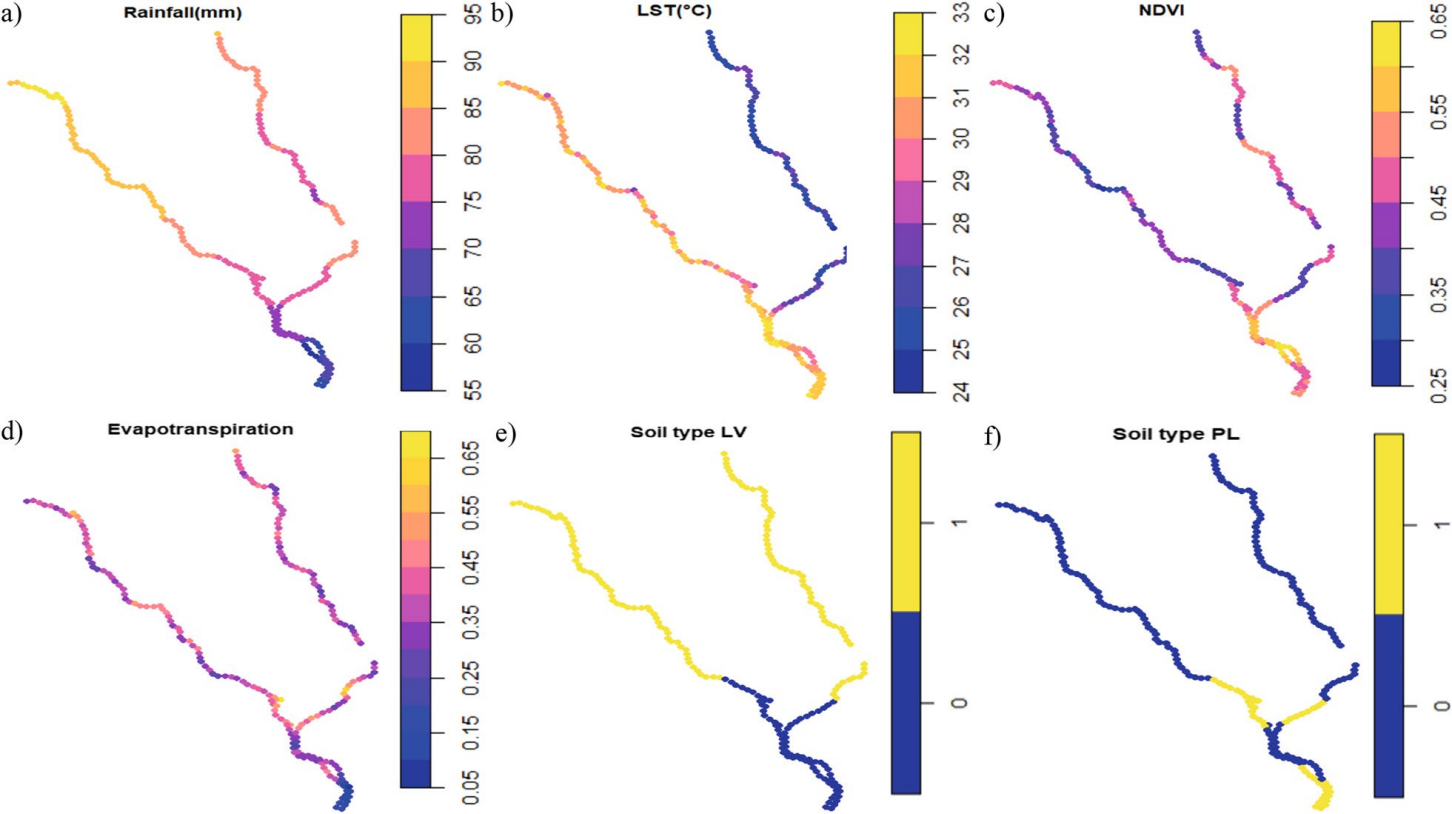
Environmental data values extracted for each prediction point. **a** Daytime land surface temperature (LST) (°C), **b** rainfall (mm), **c** evapotranspiration, **d** Normalized Difference Vegetation Index (NDVI), **e** Luvisolic (LV) soil type, **f** Planosolic (PL) soil type. **e** and **f** are compared with Gleysolic (GL) soil type. Gap in shoreline is due to the removal of CM soil type

The extracted environmental data prediction point ranges were: rainfall (59.82–90.37mm), LST [24.68–32.46 (°C)], NDVI (0.29–0.61) and evapotranspiration (0.10–0.66) along the prediction points of the shoreline. Evapotranspiration was lowest and NDVI highest along the River Shire, with the eastern shoreline having the most rainfall and lowest LST (°C) compared to the western shoreline. Luvisolic (LV) soil type was absent around the River Shire compared to Gleysolic (GL) soil type; Planosolic (PL) soil type was present at the entrance to the River Shire and south of the River Shire compared to GL soil type. The distributions of the values of the environmental covariates in Fig. 4 can be viewed in Additional file 5: Fig. S2.

### Covariance function comparison

As shown in Additional file 6: Fig. S1, the exponential quadratic covariance function was found to over-fit the model (smooth out the snail abundance excessively), and the Matérn (*κ* = 1.5) smoothed the results, whereas exponential covariance function was the roughest fit of the model. Furthermore, there seemed to be no difference in predicted $\text{log}({\widehat{\mu }}_{i})$ against distance along the shoreline for either *Biomphalaria* sp. or *Bulinus* spp. as shown in Additional file 6: Fig. S2. This suggests that the effect of the covariates (environmental data) is more prominent than in the Gaussian process.

### Model fit

The Bayesian log-linear Gaussian process model converged well according to the trace plots of the estimated parameters, and the priors were appropriately selected as shown in Additional file 7: Fig. S1 and Additional file 8: Fig. S1.

### Covariate effects

**Fig. 5:**
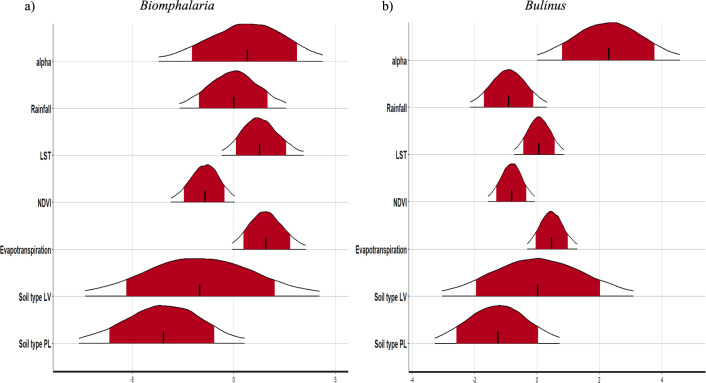
Posterior plot for each species **a** *Biomphalaria* sp.; **b** *Bulinus* spp. Red shaded area represents the 80% credible intervals (CrI) and the extent of the curve is the 95% CrI

Figure 5 shows the posterior distributions for the environmental covariate effects (on the log scale) for each species of snail, with mean snail abundance at location *i* on the x axis, with 95% CrI filled. As shown in Fig. 5 and Table 1, a significant result was reported for NDVI, where 1-SD increase in NDVI had a − 0.83 (CI − 1.57, − 0.09) reduction in the log *µ*_*i*_, mean *Bulinus* spp. snail abundance at location *i*.

**Table 1:** Estimated parameter values, mean and CrI

Parameter	*Biomphalaria*		*Bulinus*		
	Mean	95% CrI	Mean		95% CrI
α	0.46	[− 3.98,4.40]	2.32		[3.69 ${e}^{-03}$,4.56]
Rainfall	− 0.05	[− 2.74,2.75]	− 0.88		[− 2.15,0.33]
LST	1.30	[− 0.56,3.40]	0.04		[− 0.72,0.81]
NDVI	− 1.42	[− 3.09,0.10]	− 0.83		[− 1.57,− 0.09]
Evapotranspiration	1.61	[− 0.04,3.56]	0.46		[− 0.34,1.28]
Soil type LV	− 1.51	[− 7.13,4.08]	− 0.04		[− 3.07,3.11]
Soil type PL	− 3.48	[− 7.56,0.52]	− 1.29		[− 3.29,0.71]
Soil type GL					

*CrI* credible interval, *LST* land surface temperature, *NDVI* Normalized Difference Vegetation Index, *LV* Luvisols, *PL* Planosols, *GL* Gleysols

All other covariates were not significant; however, the following were still found of interest: For a 1-SD increase in the NDVI, the log *µ*_*i*_ mean *Biomphalaria* sp. abundance changes by − 1.42 (CrI − 3.09, 0.10) (reduction). For a 1-SD increase in rainfall, the log *µ*_*i*_ mean *Bulinus* spp. abundance changes by − 0.88 (CrI − 2.15, 0.33) (reduction). For a 1-SD increase in LST, the log *µ*_*i*_ mean *Biomphalaria* sp. abundance changes by 1.30 (CrI − 0.56, 3.4) (increased). For a 1-SD increase in evapotranspiration, the log *µ*_*i*_ mean *Biomphalaria* sp. abundance changes by 1.61 (CrI − 0.04, 3.56); similarly, the log *µ*_*i*_ mean *Bulinus* spp. abundance changes by 0.46 (CrI − 0.34, 1.28) (increased). For an increase of PL soil type compared to the baseline GL soil type, the log *µ*_*i*_ mean *Biomphalaria* sp. abundance changes by − 3.48 (CrI − 7.13, 0.52) (reduction); similarly, the log *µ*_*i*_ mean *Bulinus* spp. abundance changes by − 1.29 (CrI − 3.29, 0.71) (reduction). No association could be found for LV soil compared to GL soil type.

### Model predictions

**Fig. 6:**
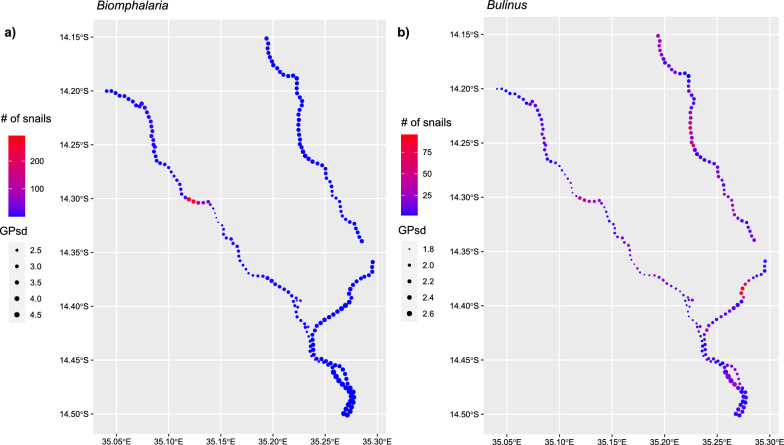
Two-dimensional mean Gaussian process (GP) prediction of number of snails $\text{log}({\widehat{u}}_{i})$ along the shoreline (km). **a** *Biomphalaria* sp. **b** *Bulinus* spp. Legend: Blue to red stands for exponential of mean GP—number of snails. Dot size represents the standard deviation of the posterior predictive distribution at each vertex

For *Biomphalaria* sp., we predicted the greatest number of snails present to be close to Moet and Koche schools. For *Bulinus* spp., a higher number of snails was predicted over a wider area, close to Moet, Koche, Mtengeza, Chipeleka and Sungusya schools. However, for both *Biomphalaria* sp. and *Bulinus* spp., there was great uncertainty around all locations (2D version, Fig. 6; 1D version, Additional file 9: Fig. S1).

## Discussion

**Fig. 7:**
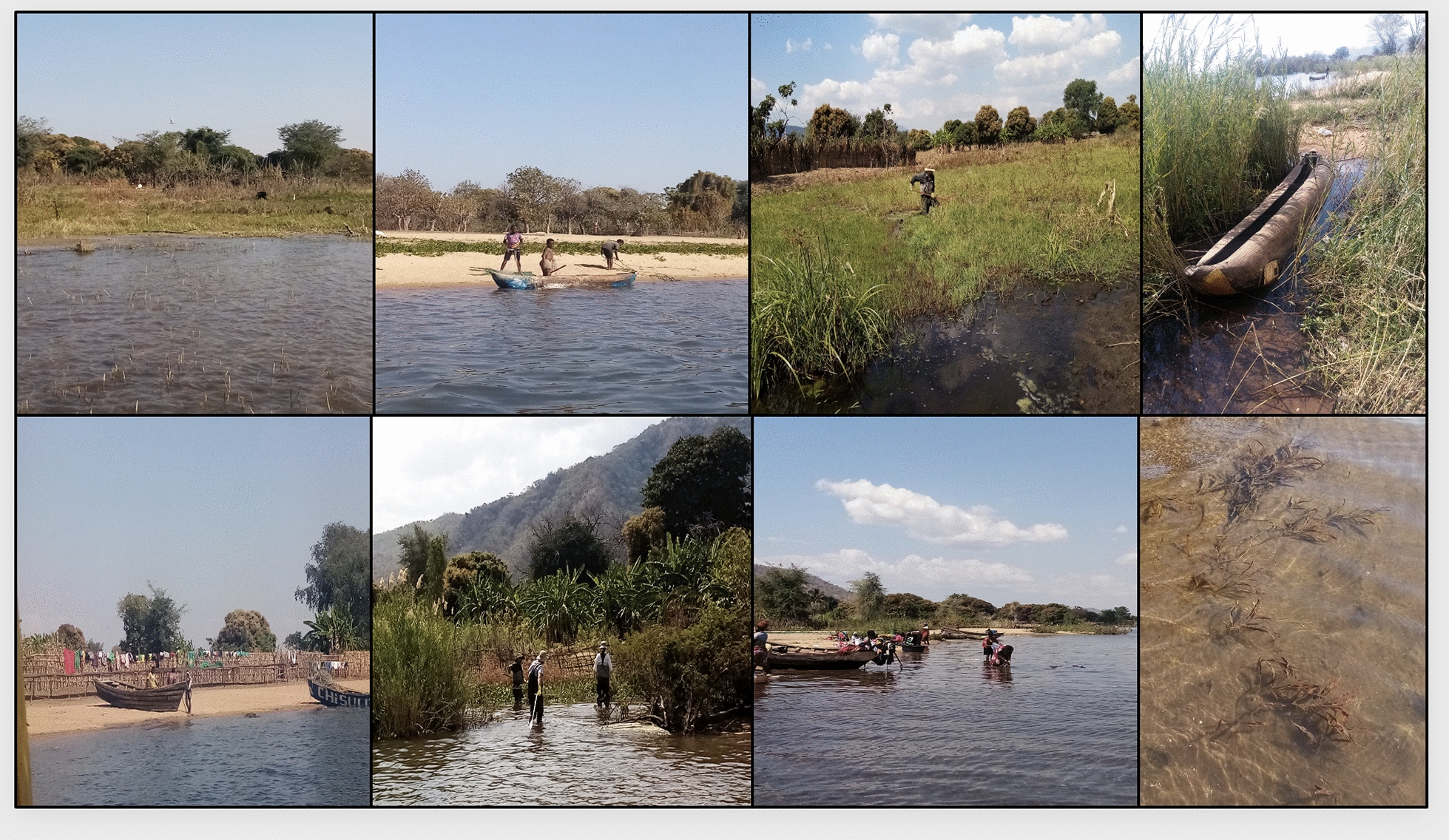
A collection of location photographs representative of the variation of the Southern part of Lake Malawi. Pictures taken during field work studies carried out during August 2022 showing the east side of the southern part of the Lake Malawi shoreline (unpublished). Pictures taken by Alexandra Juhasz

Our secondary spatial analysis has made a seminal attempt to analyse, interpolate and then predict *Biomphalaria* and *Bulinus* snail distribution in unsampled locations in the southern part of Lake Malawi, Mangochi District. Our study found a significant negative association between NDVI and snail abundance for *Bulinus* spp. Analysis of our results are also indicative of a similar association between NDVI and *Biomphalaria* sp. abundance, although this was not significant given our currently available data. Other covariates considered in the model were all non-significant, as reported in Table 1; despite their uncertainty, we reported an increase in rainfall along the shoreline, which causes a reduction in the mean snail abundance found along the shoreline for *Bulinus* spp. However, an increase in evapotranspiration and in LST along the shoreline may each cause an increase in the mean snail abundance found along the shoreline for both *Bulinus* spp. and *Biomphalaria* sp. For soil type, we found that an increase in PL or LV caused a reduction in the mean abundance found along the shoreline compared with GM. The characteristics of the shoreline of the southern part of Lake Malawi are known to vary considerably over focal areas (Fig. 7) and in turn can increase or decrease snail abundance. We discuss our findings below upon consideration of other studies and establish how this could help to identify risk of schistosomiasis transmission risk locally.

In most previous studies, increasing vegetation (higher NDVI) was shown to have a positive association with snails found due to vegetation providing more suitable breeding sites, whereas our study suggests a negative association [31–33]. This difference in result is likely due to our focus on Lake Malawi, where molluscivorous fish may be present, as opposed to a more general area including smaller bodies of stagnant water, which typically lack such predatory fish.

The presence of land vegetation around the shoreline may well be descriptive of the land topology and hence the depth of the water in the immediate vicinity—deeper water is likely less conducive to snail habitats because of the absence of aquatic flora. Furthermore, the type of vegetation and whether it is submerged or nonemergent floating vegetation are known to be important as the freshwater snails need protection from wave action and food resources, aiding egg-laying, and this was not considered in our model [34, 35].

There is an indication that an increase in rainfall decreases snail abundance in our model despite its uncertainty. First, this result could be due to the water flow increasing and spreading to new locations, disrupting freshwater snail habitats [32]. Second, an increase in rainfall has been reported to increase turbidity of water and in turn decrease the presence of snails (through disrupting their habitat) [32, 33]. Lastly, increases in rainfall and water flow have also been reported to cause rapid changes in temperature causing thermal shock and reduced egg-laying of the freshwater snails, causing an overall reduction in snail abundance [35].

In contrast to our result, some cases studies have found increase in snail abundance during increase in rainfall. For instance, when excess rainfall, known as flooding, occurs, new areas of snail habitat can occur where previously snails were not present or eliminated. Runoff water can create new pools adjacent to the shoreline or inland, allowing more breeding sites to be colonized by the intermediate snail host and thus increasing freshwater snail abundance [5]. Consequently, flooding can change the human-snail contact interplay, through an indirect effect on human behaviour, and thus the associated risk of schistosomiasis transmission [36, 37]. However, other studies have suggested that during flooding these newly established pools of water can lead to humans visiting these new sites instead of the Lake Malawi with a possible decreased likelihood of snails being present already in these new sites, which could lead to a reduction in schistosomiasis transmission [31, 36]. Adding to the complexity, rainfall and water levels are known to oscillate over time, with a general decrease in lake levels reported more recently, with ongoing localised peaks of lake levels occurring through time [2]. This could impact the snail abundance and its presence spatially and temporally and indirectly affect human behaviour as mentioned before [2, 36, 37], for example, if the lake levels are regulated by needs for hydroelectricity or because many individuals prefer to make contact with shallower and more safe areas of the lake [38, 39].

Analysis of our results suggest that an increase in LST increases *Biomphalaria* sp. and *Bulinus* spp. snail abundance. Many laboratory studies have been carried out to determine the optimal temperature for snail survival. For *Biomphalaria* sp. snails the optimum temperature has been found to between 15 and 30 °C, where there is a decrease in snail abundance above 30 and 35^*◦*^*C*, and no snails survive above 35 °C [37, 38]*.*For our prediction points along the shoreline, the LST ranged between 25 and 32 °C, which suggests *Biomphalaria* sp. snail abundance still increases above 30 °C. This difference could be due to it being in a natural environment where snails are able to adapt to climate change [40]. It has also been reported that freshwater snails move further into the lake when temperatures increase, which we did not consider in our model because it was constrained to the shoreline and buffer area [41].

Similarly, there is an indication that increased evapotranspiration increased the *Biomphalaria* sp. and *Bulinus* spp. snail abundance in our study. The increase in evapotranspiration, also known as the increase in evaporation of water, is known to have an impact on pH, salinity (salt concentration), conductivity and temperature of water through unpublished field studies; these finer physical characteristics need to be further investigated. This suggests an increase in evapotranspiration causes these unexplored covariates to become more habitable for intermediate snail hosts, causing an increase in local snail abundance. How these unexplored covariates interact and their effect on snail abundance are not considered in our study but have been investigated in other studies [5].

Our study found PL soil type decreases snail abundance compared to GL soil type. PL soil types are clay-based, plinthic soils with high concentrations of iron. GL soils are mineral soils, which are a mixture of sand, silt and clay. Both are muddy when rainfall occurs (become water-logged) [42]. A previous study by Koch et al. [7] found the opposite results with muddy soil being reported to improve the survivability of *Biomphalaria* sp. by preventing them from losing moisture in the hot and dry seasons compared with sandy ones and stony and decomposing material [7]. The difference between PL and GL soils is that GL is known for its iron reduction [43]. Kulina et al. [44] reported an increase in transmission of snail risk in groundwater with higher iron concentration [44]. We found a different result, which suggested another chemical within the soil type could be interacting with the snail abundance and affecting transmission. Furthermore, there was uncertainty in our results. The soil types from SOTER database are for wide scales; lower level data are needed to improve the information on more localised soil types [29]. Other resources have been created, for example SoilGrids for Africa, which, if time permitted and it provided lower level data for southern Malawi, could be applied to our study in the future [45].

Our secondary analysis study shows substantive heterogeneities in snail distributions along the lake’s shoreline, with certain schools being close to areas of increased abundance of snails. Hence, SAC attending these schools may be more likely to be exposed to schistosomiasis. Moet and Koche schools were predicted to be nearest to the highest number of *Biomphalaria* sp. present along the shoreline, suggesting that more *S. mansoni* infections probably occur at these schools compared to the 10 other schools. Whereas, Moet, Koche, Mtengeza, Chipeleka and Sungusya were all predicted to be nearest to the highest number of *Bulinus* spp. However, for both *Biomphalaria* sp. and *Bulinus* spp., predicted presence along all the shoreline had large uncertainty. Furthermore, we cannot be certain about the exposure risk for the SAC as this secondary analysis does not consider their water contact patterns, including where they visit (how far they travel to) the shoreline, frequency, type of contact and how long they remain at the shoreline. This needs to be further investigated as previous studies have reported increased snail abundance in localised areas where more water contact is occurring [41, 46]. In addition, the ability to measure exposure risk for SAC from our secondary analysis is dependent on presence of snails in an area being indicative that freshwater snails present are shredding cercariae, but it is difficult to be certain of this [35].

There are many more physical, chemical and environmental factors (abiotic and biotic) which could impact *Schistosoma* intermediate snail habitats and their relative snail abundance; these were not considered in our model because of time constraints or non-accessible data, e.g. pH, salinity, conductivity, flow velocity, turbidity, calcium and bicarbonate concentration, dissolved oxygen, soil density and water capacity [35, 36, 47]. Furthermore, other factors such as food source, pollution (e.g. discarded plastics), parasitism and even the competition for snail habit with other organisms within an area were not considered in our model [47]. Variation in human movement patterns can make it difficult to locate the location of acquired infected. A land use and human influence index could have been included in our model if time had permitted [48].

One limitation of our study is the restricted study period (November 2017 to June 2019, except for the 5-year evapotranspiration time frame) as well as taking the mean values for each prediction location. Rabone et al. [41] reported seasonality affecting snail abundance, with higher snail abundance during the dry season compared to the wet season. For instance, seasonality can affect growth of vegetation and therefore the freshwater snail's life due to the variation in sunlight, therefore leading to changes in snail abundance [47]. In the future, we would like to investigate how seasonality affects the snail distribution using our model. We reported on the seasonal changes of the covariate data in Additional file 4: Fig. S1, Fig. S2 and Fig. S3; this allows observation on how covariate data change over time, although this was not considered in our model.

As mentioned before, another known limitation is that snails are not only found on the shoreline of Lake Malawi but also in pools adjacent to the lake or rivers, ponds and streams. This has been reported to affect snail abundance by affecting the microhabitat, for instance by changes temperatures [41, 49]. Unpublished field work studies in 2021 showed that on southern lake slopes in areas, the western side of the shoreline had longer shallower areas. The area near the Upper Shire River is known to have more vegetation and swampy areas than the rest of the shoreline. Bathymetric data for water depth were originally considered in our secondary analysis, taken from the GLObal Bathymetric (GLOBathy) dataset, which relies on HydroLakes dataset [49]. However, it was excluded from the study because of missing River Shire values as shown in Additional file 10: Fig. S1, Fig S2 and Fig S3. Therefore, water depth needs to be further investigated. As mentioned before, water levels are known to vary over time, leading to changing water depth. *Bulinus* spp. and *Biomphalaria* sp. have different preferences regarding water depth and vegetation [1–3].

An important main limitation of our analysis is the resolution of the raster data we used as covariates. Many remotely sensed metrics are known to be inaccurate over water. Therefore, we positioned our shoreline linestring just inland of the water’s edge. Thus, any associations between land-based measurements and habitat conditions in the water are likely to only indirectly affect snail abundance. A repeat study, using direct observations of shoreline habitat composition, perhaps using towed arrays of sensors behind a boat or done directed close to the water’s edge, may be able to provide a more accurate map of predicted snail abundance.

## Conclusions

Our study provides a preliminary method of predicting the abundance of *Biomphalaria* sp. and *Bulinus* spp. snails along the shoreline of Lake Malawi, given malacological data collected at sparse locations and remotely sensed environmental data. Furthermore, our study shows substantive heterogeneities in snail distributions along the lake and abundance information which may be used to develop further statistically grounded study designs to improve the identification of likely snail habitats posing a high risk for schistosomiasis transmission.

### Supplementary Information

Additional file 1. Dataset.

Additional file 2. Figure S1, Figure S2.

Additional file 3. Model formulation.

Additional file 4. Figure S1, Figure S2 and Figure S3.

Additional file 5. Figure S1 and Figure S2.

Additional file 6. Figure S1 and Figure S2.

Additional file 7. Figure S1.

Additional file 8. Figure S1.

Additional file 9. Figure S1.

Additional file 10. Figure S1, Figure S2 and Figure S3.

## Data Availability

All code for this publication is accessible on Zenodo 10.5281/zenodo.10410622. The primary data are provided in Additional file 1: Dataset.

## Abbreviations

BMLM: Bayesian multilevel model
CM: Cambisols
°C: Degree Celsius
GP: Gaussian process
GL: Gleysols
GLOBathy: GLObalBathymetric
IS: Intestinal schistosomiasis
ISRIC: International Soil Reference and Information Centre
ITCZ: Inter-Tropical Converge Zone
LPDAAC: Land Processes Distribution Active Archive Center
LST: Land surface temperature
LV: Luvisols
MCMC: Markov chain Monte Carlo
Millimetre: Mm
MODIS: Moderate-resolution imaging spectroradiometer
NDVI: Normalised Difference Vegetation Index
1D: One-dimensional
PL: Planosols
SAC: School-aged children
SOTER: Soil Terrain Database for Malawi
TAMSAT: Tropical Applications of Meteorology using SATellitedata and ground-based observations
2D: Two-dimensional
UGS: Urogenital schistosomiasis
