# Supplementary material for: A geospatial analysis of local intermediate snail host distributions provides insight into schistosomiasis risk within under-sampled areas of southern Lake Malawi
Source: Parasit Vectors. 2024 Jun 27;17:272. doi: 10.1186/s13071-024-06353-y (PMC11209974; doi:10.1186/s13071-024-06353-y)
Supplement: Supplementary file 2 — Additional file 2. Figure S1, Figure S2. [file 13071_2024_6353_MOESM2_ESM.pdf]

Additional file 2: Supplementary information

Construction of 200 prediction points along shoreline

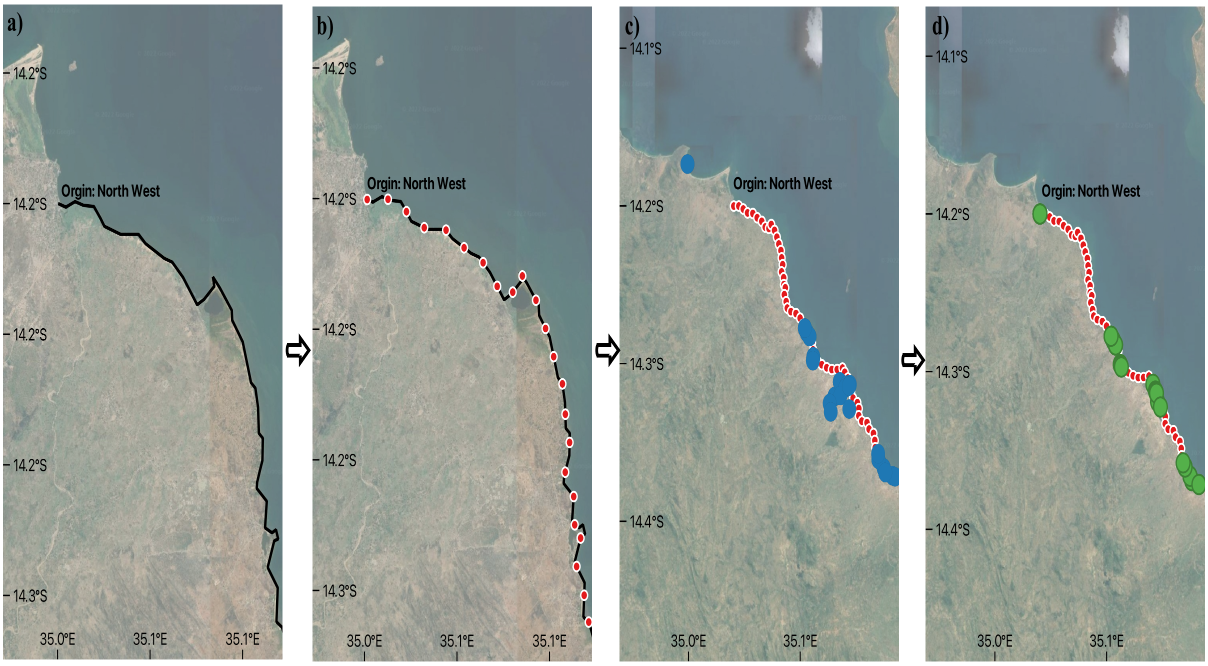

Figure S1: Flow diagram showing the stages for constructing the 200 predictions along the shoreline. **a)** A 2-D linestring was drawn by hand following the shoreline as shown by Google Satellite imagery, **b)** the linestring was re-sampled to 4000 equally spaced vertices and resampled them to 200 equally intervals (red dots), **c)** observed sampling site locations (blue dots), **d)** each observed sampling site location was snapped to its nearest vertex (green dots).

(a) *Biomphalaria* sp.

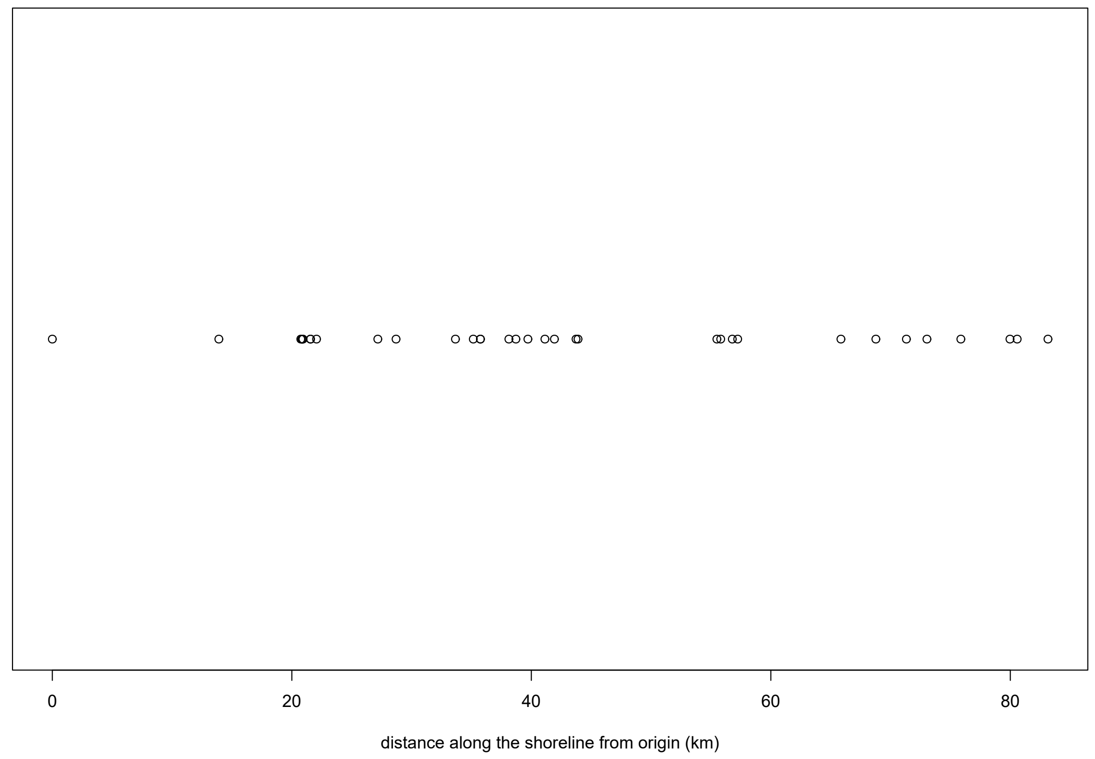

(a) *Bulinus* spp.

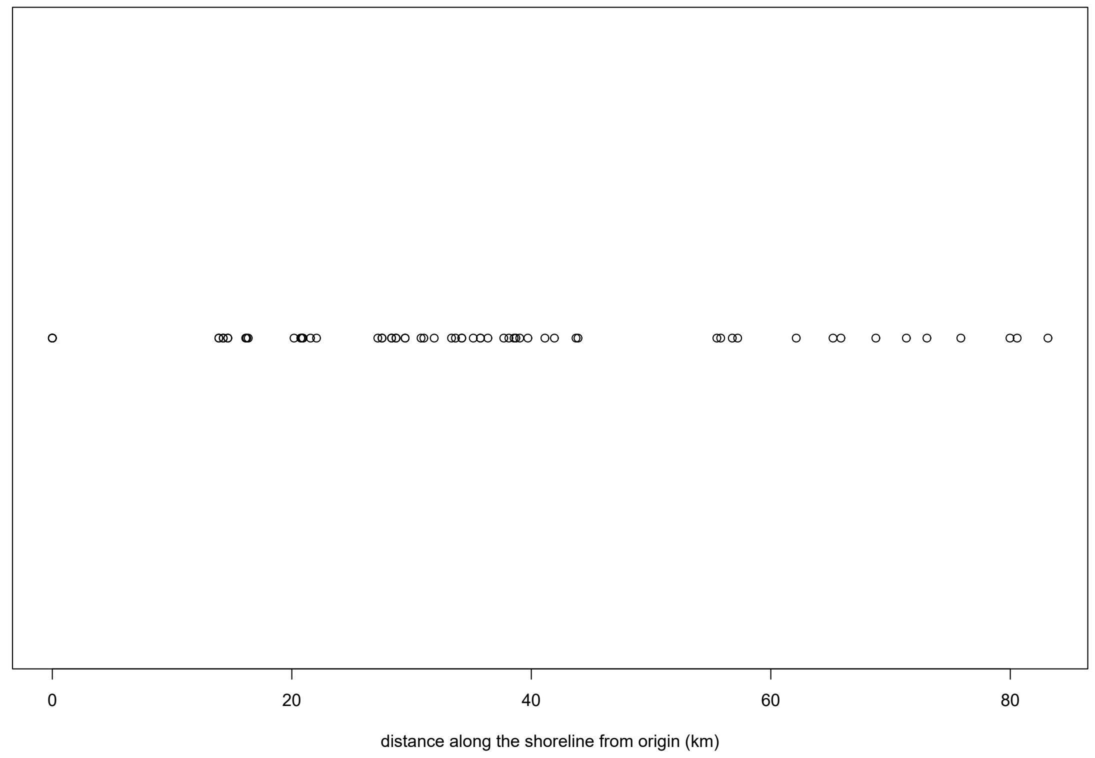

Figure S2: The distance along the line from the origin (northwest-most vertex) to each of the snapper observed sampling site locations for each species a) *Biomphalaria* sp. b) *Bulinus* spp..
