# Supplementary material for: A geospatial analysis of local intermediate snail host distributions provides insight into schistosomiasis risk within under-sampled areas of southern Lake Malawi
Source: Parasit Vectors. 2024 Jun 27;17:272. doi: 10.1186/s13071-024-06353-y (PMC11209974; doi:10.1186/s13071-024-06353-y)
Supplement: Supplementary file 3 — Additional file 3. Model formulation. [file 13071_2024_6353_MOESM3_ESM.pdf]

Additional file 3: Supplementary information

Model Formulation

Model

We used a Bayesian log-linear Gaussian Process model to smooth observations of snail abundance along the shoreline. We assume that the number of snails y_i at observed (sampling) locations x_i expressed as distance along the shoreline relative to an arbitrary reference point follow a Poisson distribution with mean μ_i:

$$y_i \sim Poisson(\mu_i),$$

The mean number of snails is then modelled on the log-scale as sum of two components: a set of covariates expressed through the design matrix Z and a Gaussian process:

$$\log(\mu_i) = \alpha + Z^T(x_i)\beta + S(x_i)$$

S is modelled using a 1-Dimensional (1D) Gaussian process with exponential correlation function with standard deviation σ and fixed length scale $\phi = 3.33\text{km}$ (5% range of measured shoreline). We also included an additional uncorrelated variance term τ (nugget effect). We place priors on our unknown model parameters,

$$\alpha \sim \mathrm{N}(0,100)$$

$$\beta_i \sim \mathrm{N}(0,5)$$

$$\sigma \sim \Gamma(1,1)$$

$$\tau \sim \Gamma(0.1,0.1)$$

Where Γ distribution is defined using shape and rate parameters. The STAN probabilistic programming language was used to fit the model, allowing us to project the Gaussian process onto a fine grid of points along the shoreline to create predictions of snail abundance at arbitrary points in our study region.

GP prediction

To get the GP prediction values we find the mean of the Poisson distribution variables.

$$\log(\widehat{\mu_i}) = \hat{\alpha} + z^T(x_i^*)\hat{\beta} + \hat{s}(x_i^*),$$

where $\widehat{\mu_i}$ is our GP prediction of the number of snails present, x_i^* is the covariate values for each prediction point, where i=1,….,N point.

Latent Gaussian process

$$s(\vec{x}) \sim GP(0, \Sigma^2)$$

Where the mean, 0 and Σ is the covariance matrix. The exponential covariance function is represented as the following:

$$\Sigma_{ij}^2 = \begin{cases} \sigma^2 e^{\frac{-\|x_i - x_j\|}{\sigma}} & if\ i \neq j \\ \sigma^2 + \tau^2 & if\ i = j \end{cases}$$

Where the covariance function uses the standard deviation σ with exponential Euclidean distance or the added nugget effect, τ.
