# Supplementary material for: A geospatial analysis of local intermediate snail host distributions provides insight into schistosomiasis risk within under-sampled areas of southern Lake Malawi
Source: Parasit Vectors. 2024 Jun 27;17:272. doi: 10.1186/s13071-024-06353-y (PMC11209974; doi:10.1186/s13071-024-06353-y)
Supplement: Supplementary file 4 — Additional file 4. Figure S1, Figure S2 and Figure S3. [file 13071_2024_6353_MOESM4_ESM.pdf]

*Additional file 4: Supplementary information*

## 1D extracted environmental data

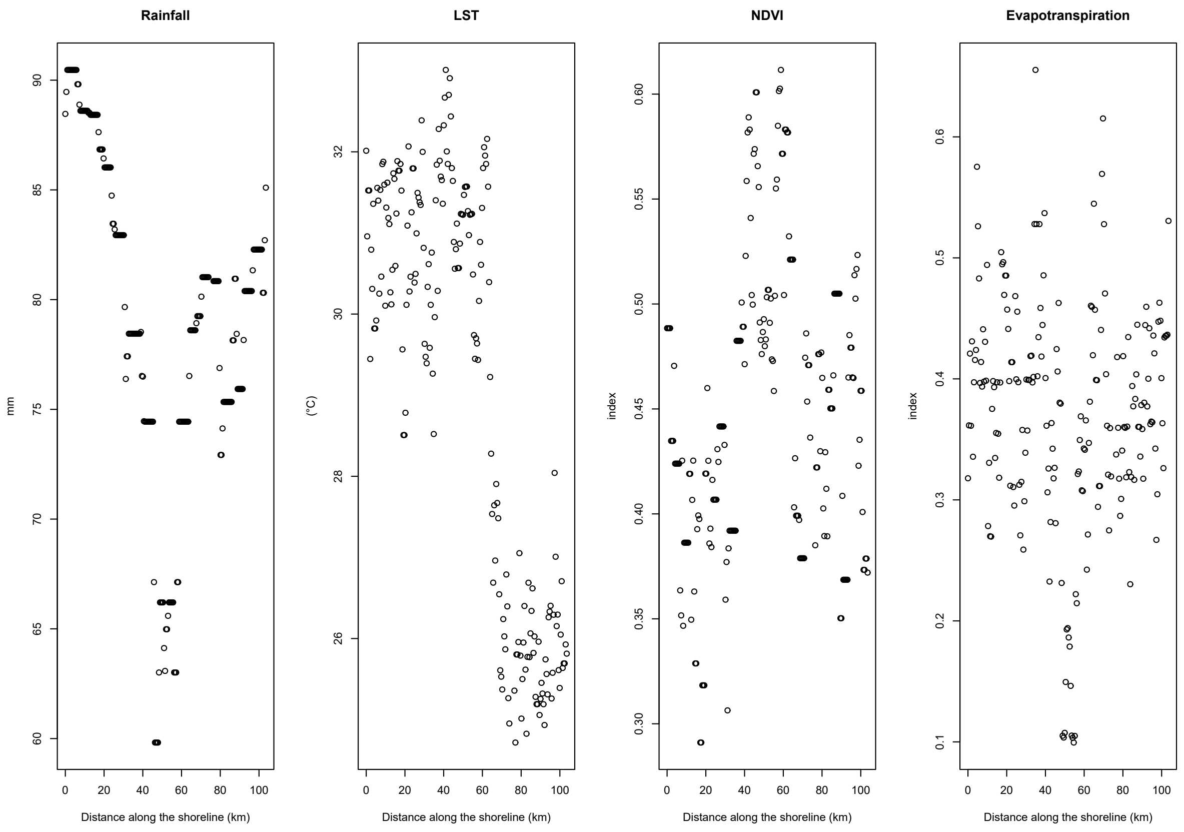


**Figure S1:** Scatterplot of environmental data versus distance along shoreline (km) for prediction points

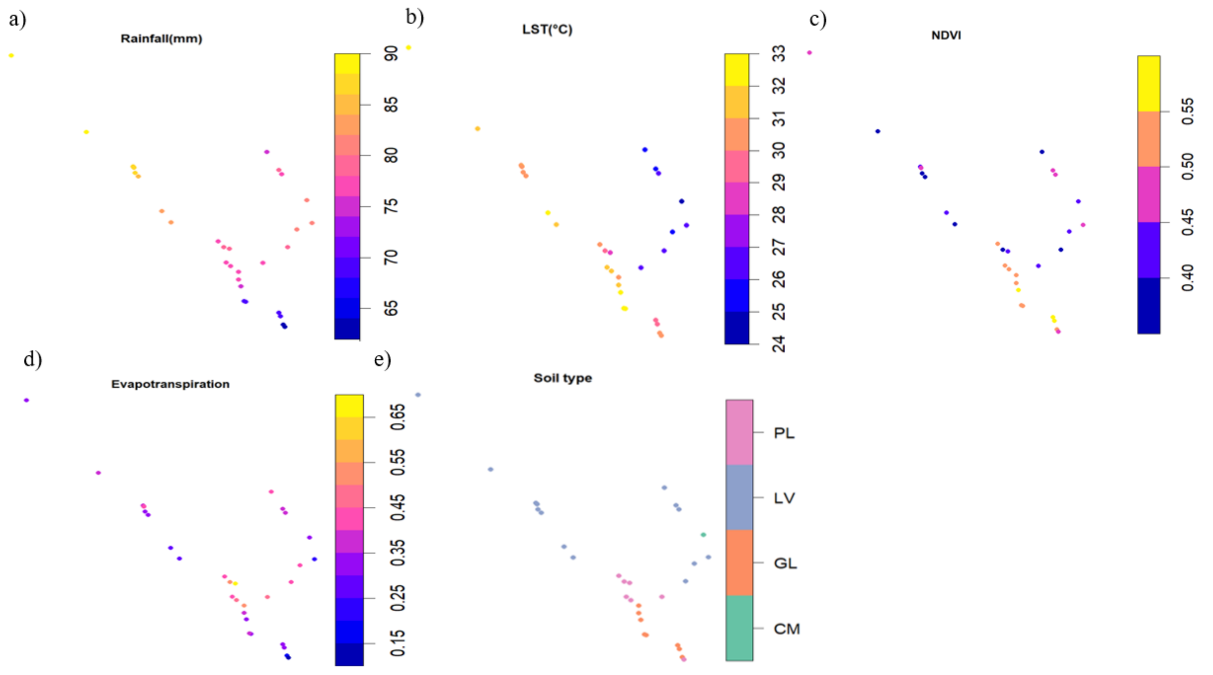

**Figure S2:** *Biomphalaria* sp. observed sample points extracted environmental data a) Rainfall (mm) b) LST (℃) c) NDVI (index) ) Evapotranspiration (index) e) Soil type

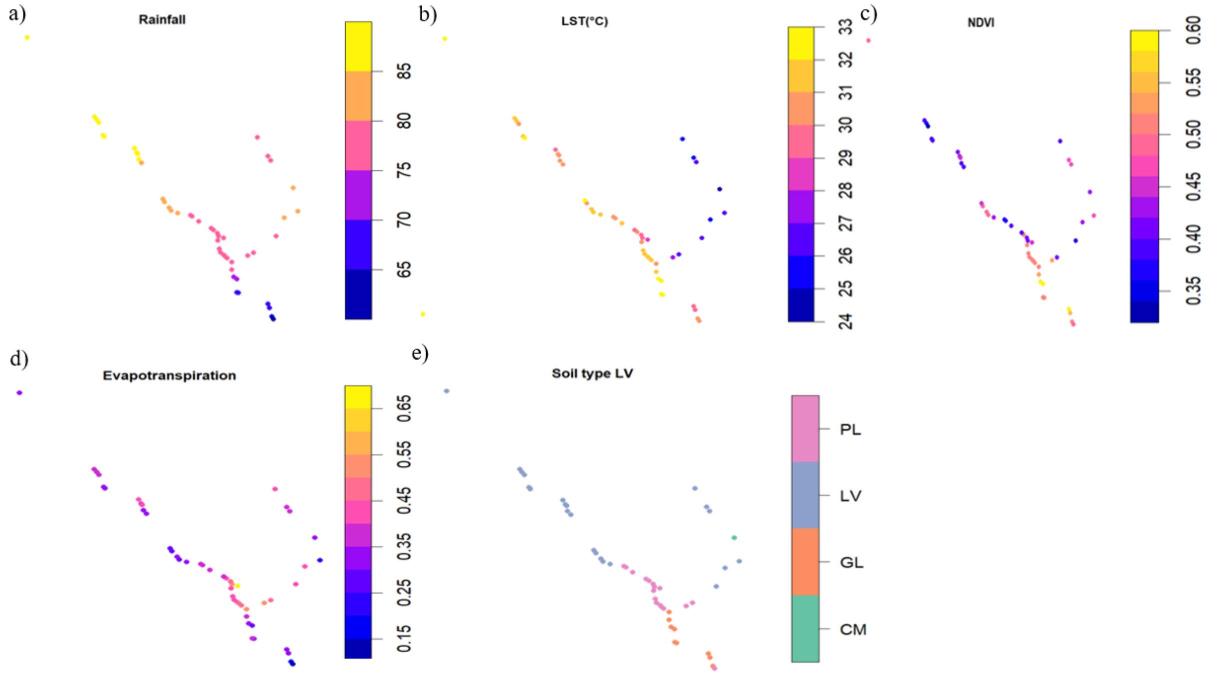


**Figure S3:** *Bulinus* spp. observed sample points extracted environmental data. a) Rainfall (mm) b) LST (℃) c) NDVI (index) ) Evapotranspiration (index) e) Soil type
