# Supplementary material for: A geospatial analysis of local intermediate snail host distributions provides insight into schistosomiasis risk within under-sampled areas of southern Lake Malawi
Source: Parasit Vectors. 2024 Jun 27;17:272. doi: 10.1186/s13071-024-06353-y (PMC11209974; doi:10.1186/s13071-024-06353-y)
Supplement: Supplementary file 5 — Additional file 5. Figure S1 and Figure S2. [file 13071_2024_6353_MOESM5_ESM.pdf]

Additional file 5: Supplementary information

Center and scaling environment data

(a) Biomphalaria sp.

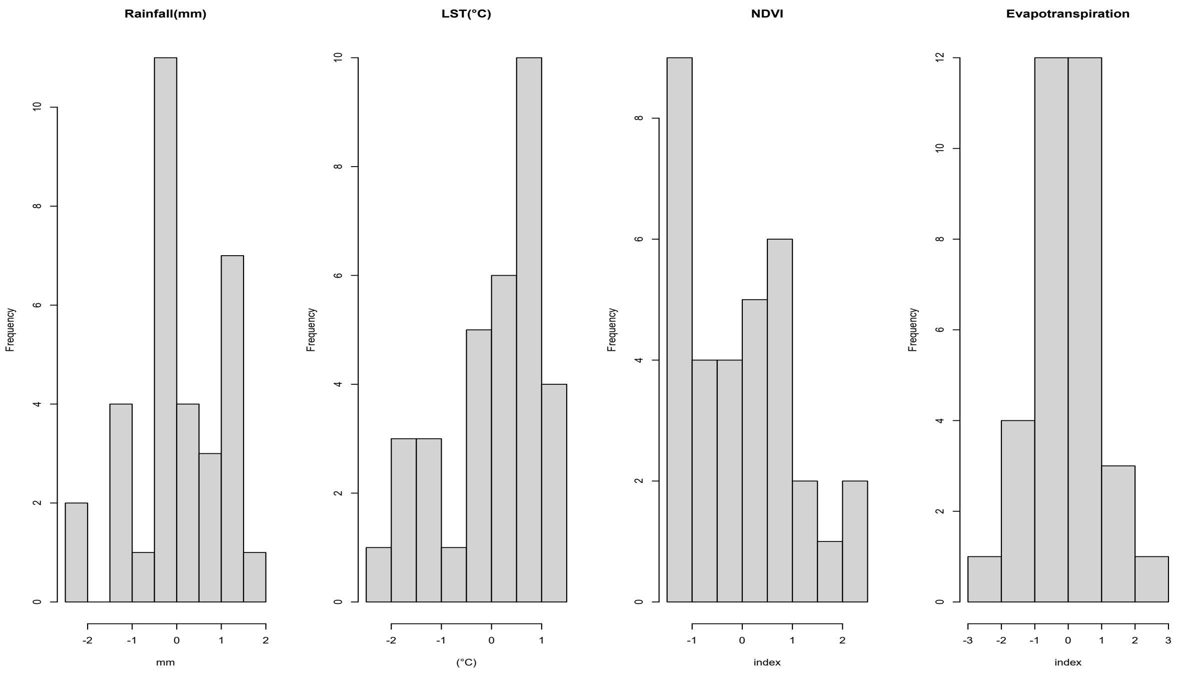

(b) Bulinus spp.

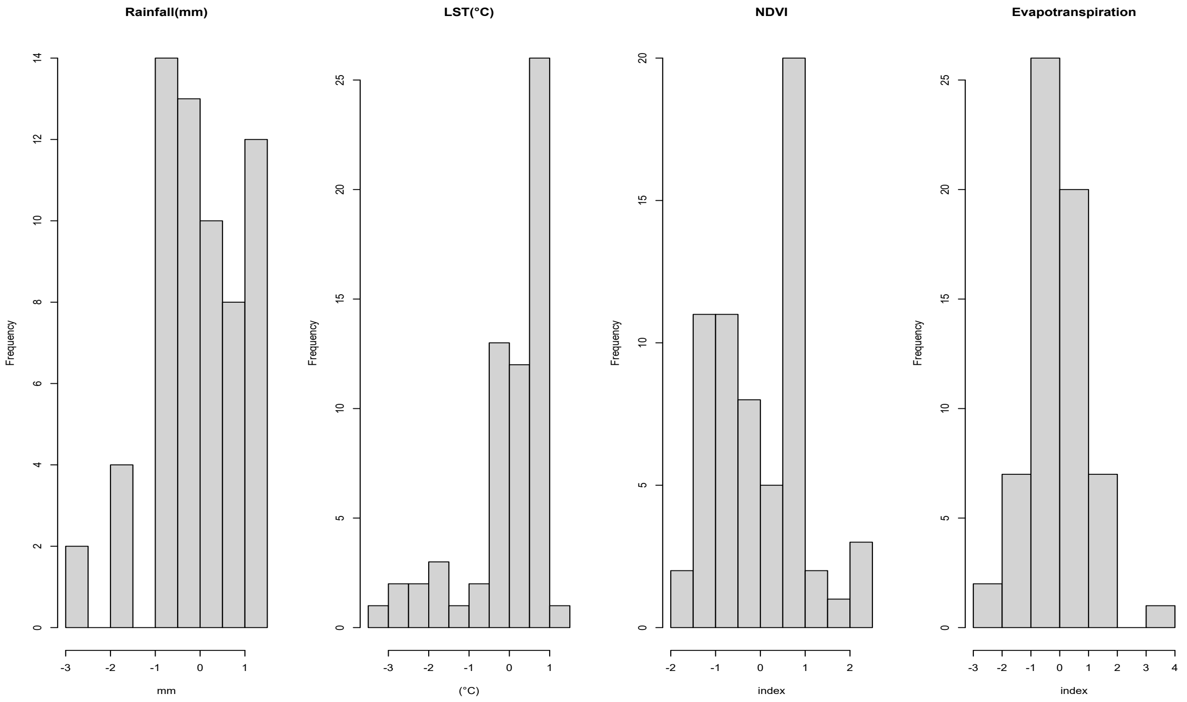

Figure S1: Observed points values for each covariate centred and scaled a) *Biomphalaria* sp. b) *Bulinus* spp.

(a) *Biomphalaria sp.*

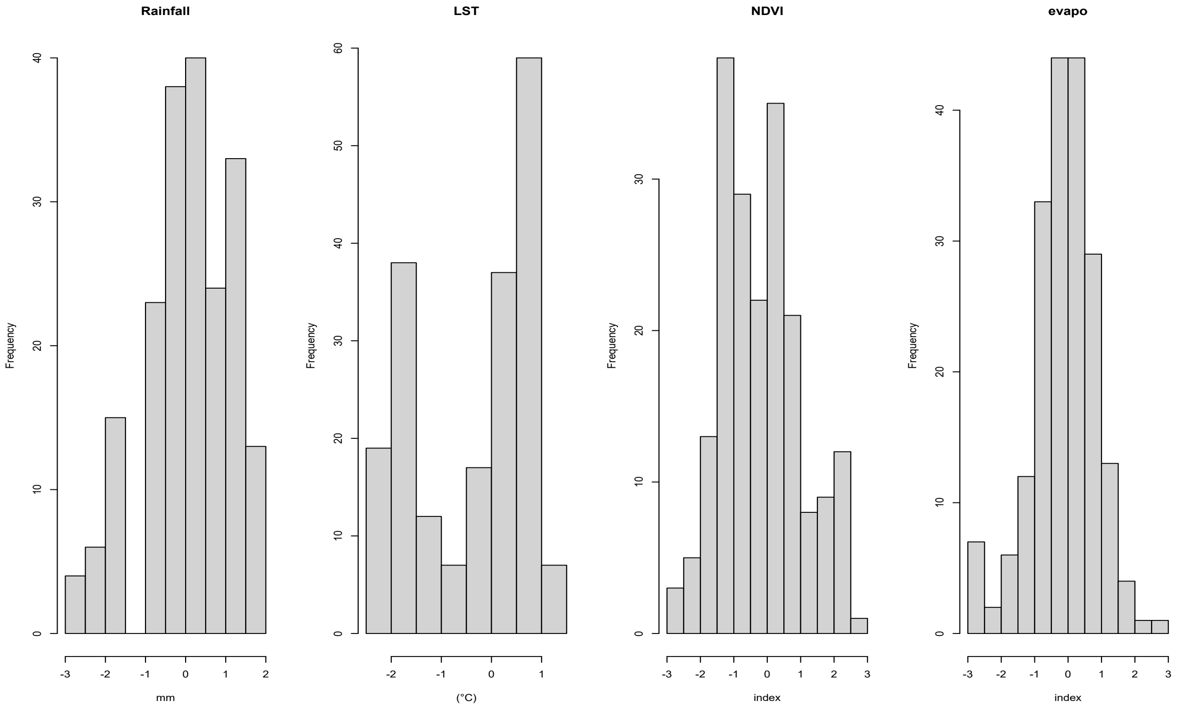

(b) *Bulinus* spp.

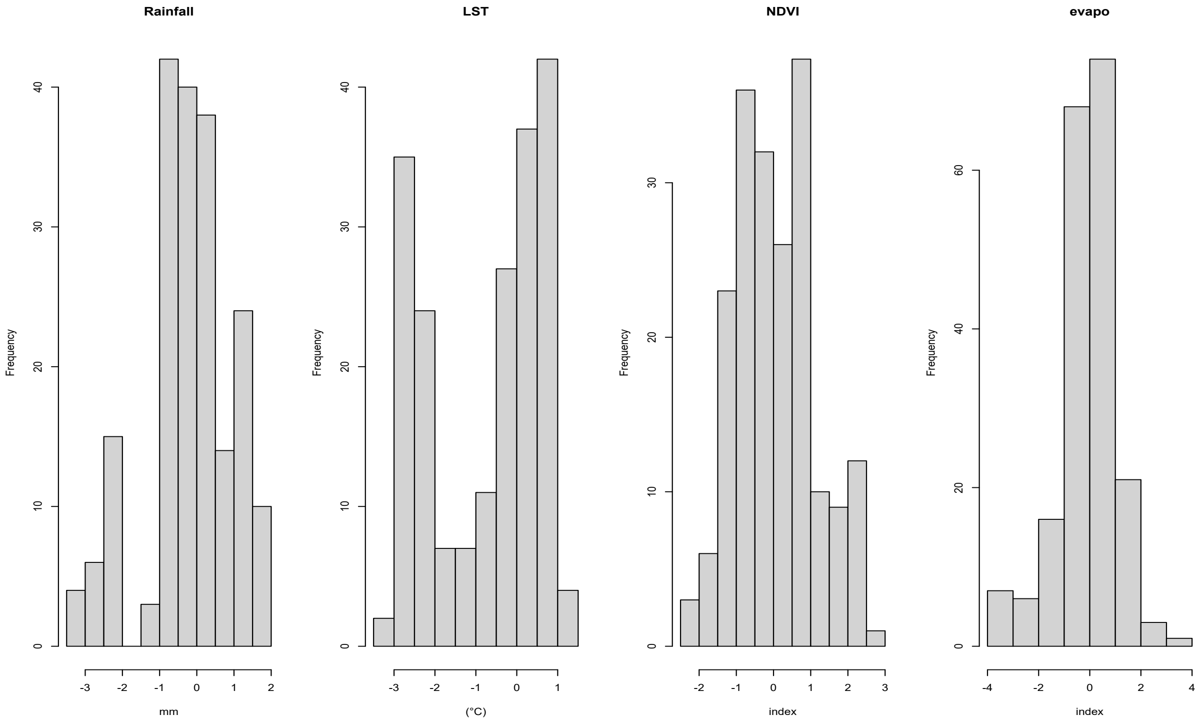

Figure S2: Prediction points values for each covariate centred and scaled. a) *Biomphalaria* sp. b) *Bulinus* spp.
