# Supplementary material for: A geospatial analysis of local intermediate snail host distributions provides insight into schistosomiasis risk within under-sampled areas of southern Lake Malawi
Source: Parasit Vectors. 2024 Jun 27;17:272. doi: 10.1186/s13071-024-06353-y (PMC11209974; doi:10.1186/s13071-024-06353-y)
Supplement: Supplementary file 6 — Additional file 6. Figure S1 and Figure S2. [file 13071_2024_6353_MOESM6_ESM.pdf]

Additional file 6: Supplementary information

Covariance functions

(a) *Biomphalaria* sp.

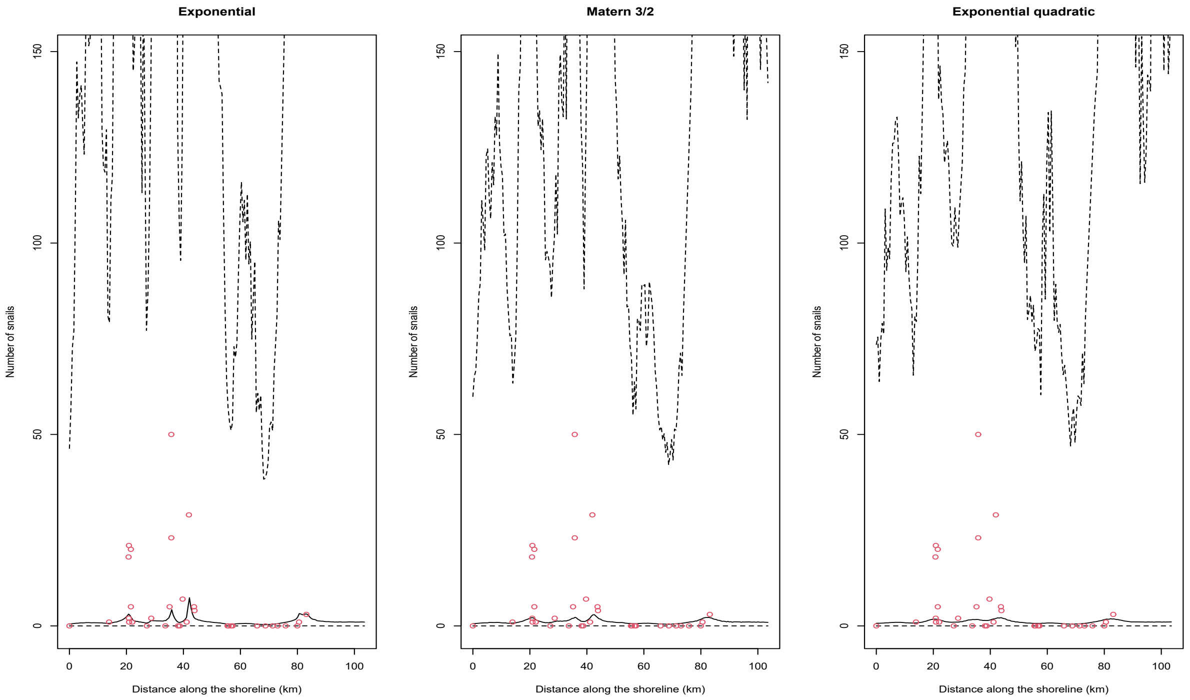

(b) *Bulinus* spp.

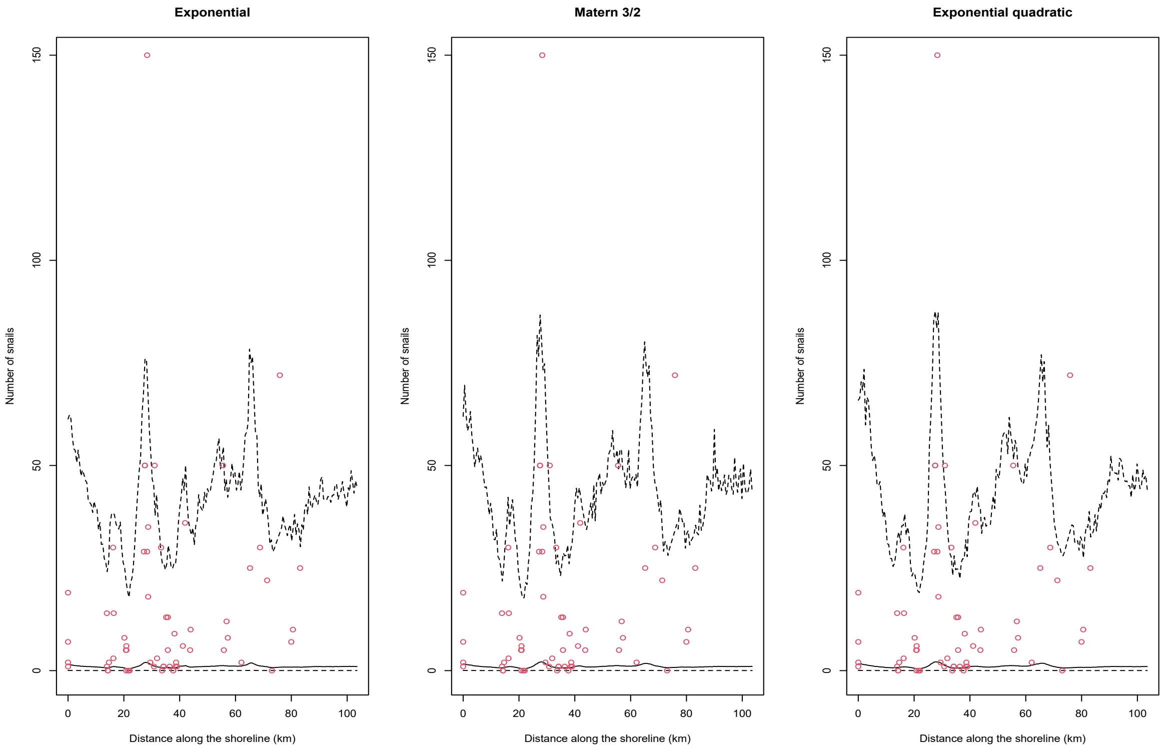

Figure S1: Comparison of covariance functions for above snails mean abundance for a) *Biomphalaria* sp. *b) Bulinus* spp. against distance along (km). Filled Straight line: Medium (50% credible intervals (Crl)) of GP prediction. Red circles: observed number of snails at sampling locations along the shoreline. Black faded lines: 95% Crl

(a) *Biomphalaria* sp.

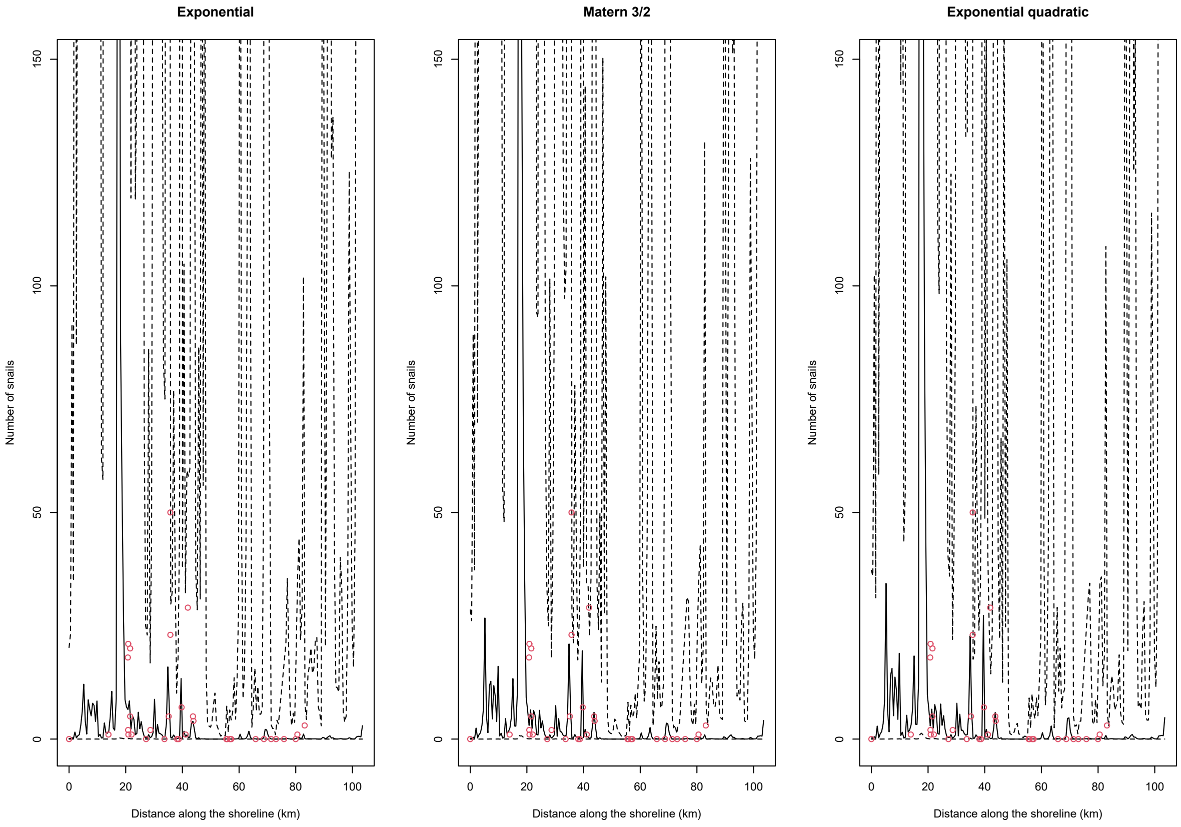

(b) *Bulinus* spp.

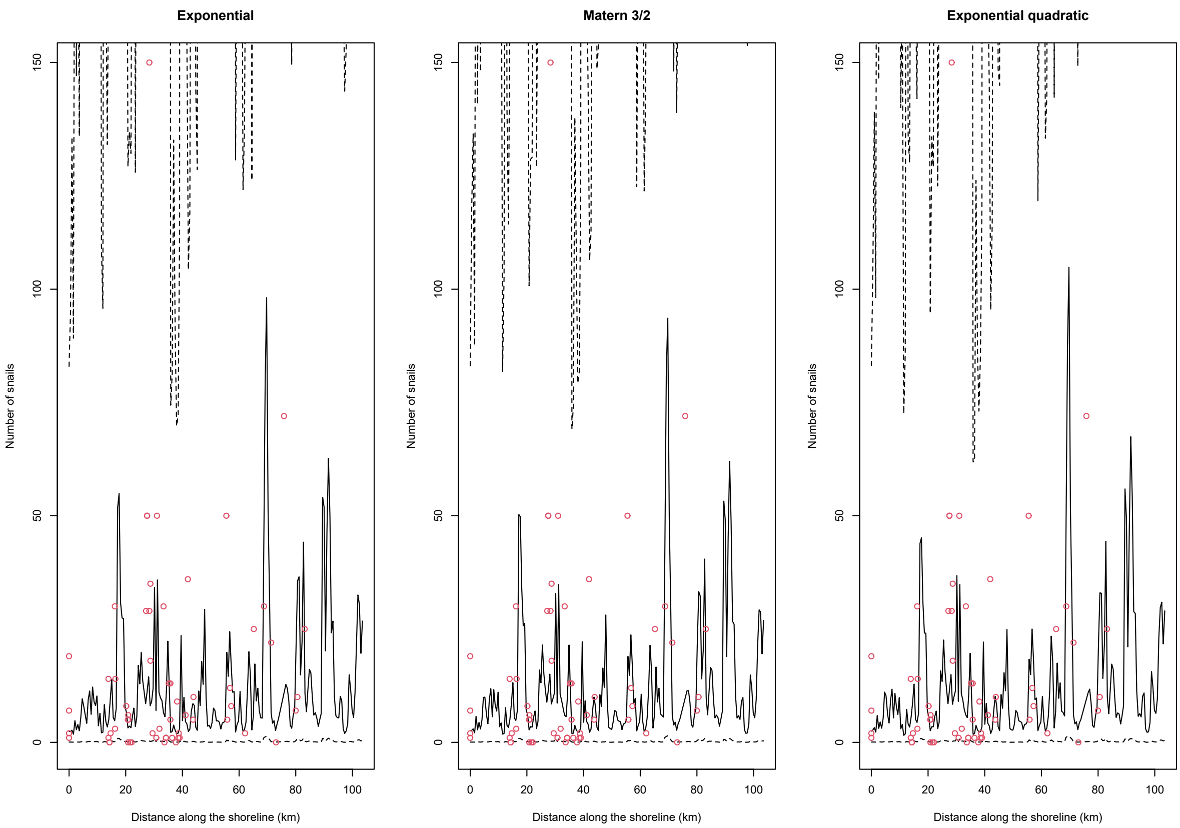

Figure S2: Comparison of covariance functions for number of snails predicted for a) *Biomphalaria* sp. *b) Bulinus* spp. against distance along (km). Filled Straight line: Medium (50% credible intervals (Crl)) of GP prediction. Red circles: observed number of snails at sampling locations along the shoreline. Black faded lines: 95% Crl
