# Supplementary material for: A geospatial analysis of local intermediate snail host distributions provides insight into schistosomiasis risk within under-sampled areas of southern Lake Malawi
Source: Parasit Vectors. 2024 Jun 27;17:272. doi: 10.1186/s13071-024-06353-y (PMC11209974; doi:10.1186/s13071-024-06353-y)
Supplement: Supplementary file 7 — Additional file 7. Figure S1. [file 13071_2024_6353_MOESM7_ESM.pdf]

Additional file 7: Supplementary information

Convergence

(a) *Biomphalaria* sp.

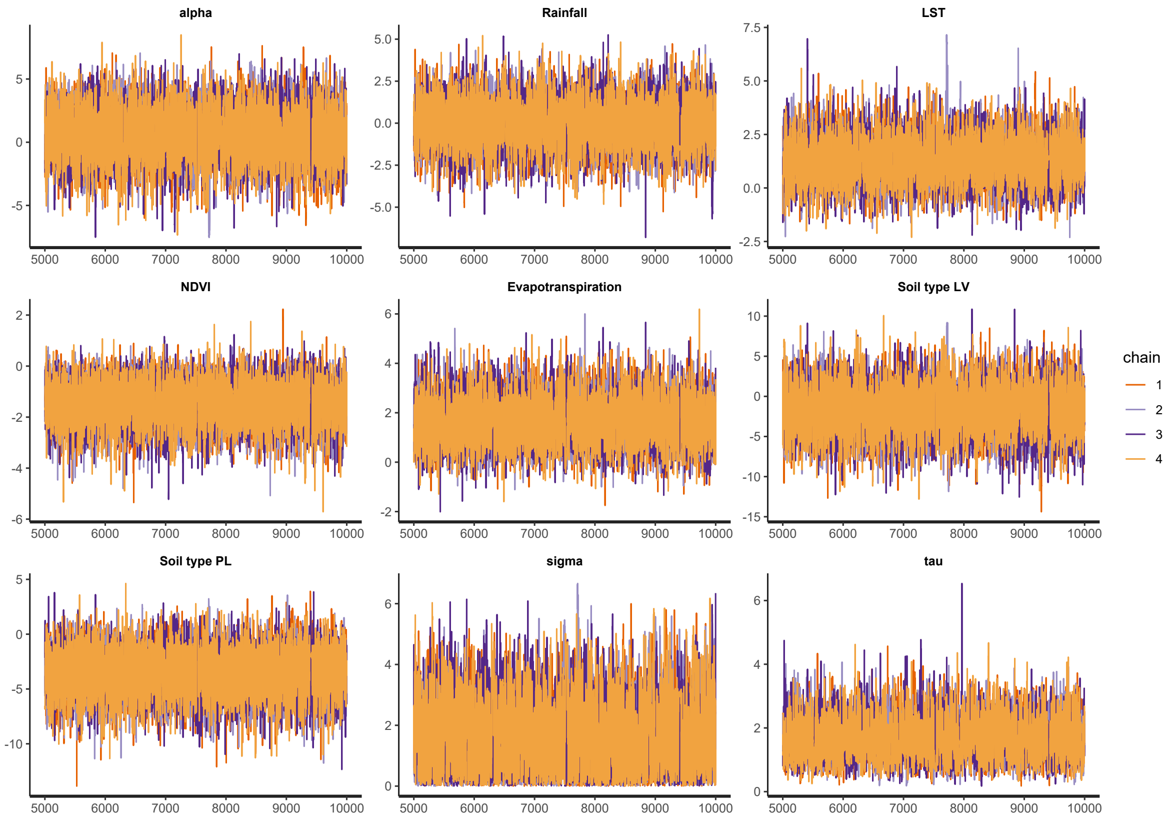

(b) *Bulinus* spp.

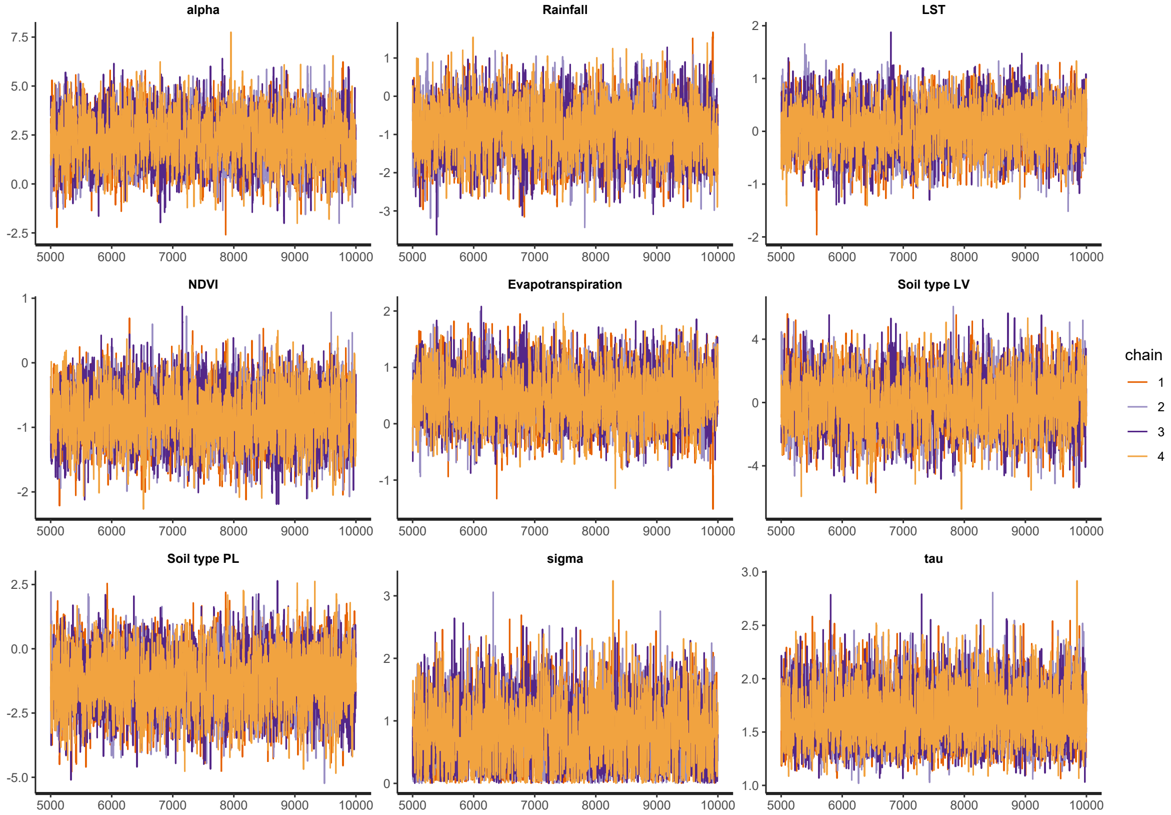

Figure S1: Trace plot of Bayesian log-linear Gaussian Process fitted model in Stan a) *Biomphalaria* sp. b) *Bulinus* spp.
