# Supplementary material for: A geospatial analysis of local intermediate snail host distributions provides insight into schistosomiasis risk within under-sampled areas of southern Lake Malawi
Source: Parasit Vectors. 2024 Jun 27;17:272. doi: 10.1186/s13071-024-06353-y (PMC11209974; doi:10.1186/s13071-024-06353-y)
Supplement: Supplementary file 8 — Additional file 8. Figure S1. [file 13071_2024_6353_MOESM8_ESM.pdf]

Additional file 8: Supplementary information

Priors and posteriors

(a) *Biomphalaria* sp.

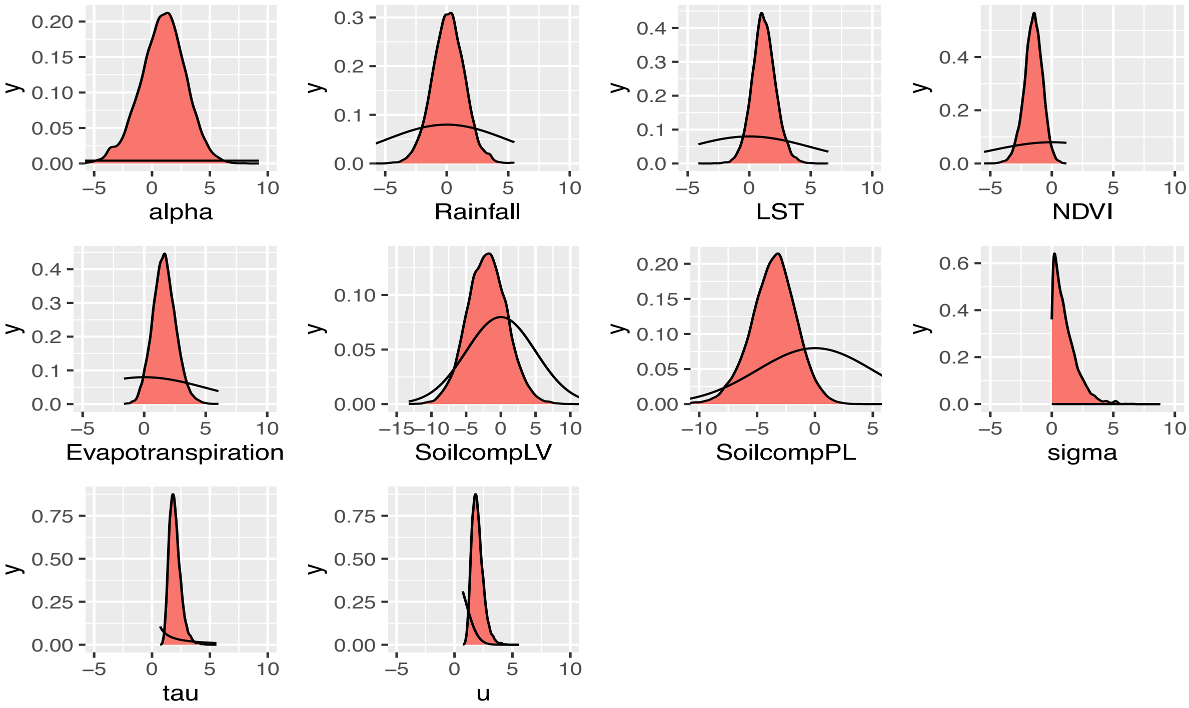

(b) *Bulinus* spp.

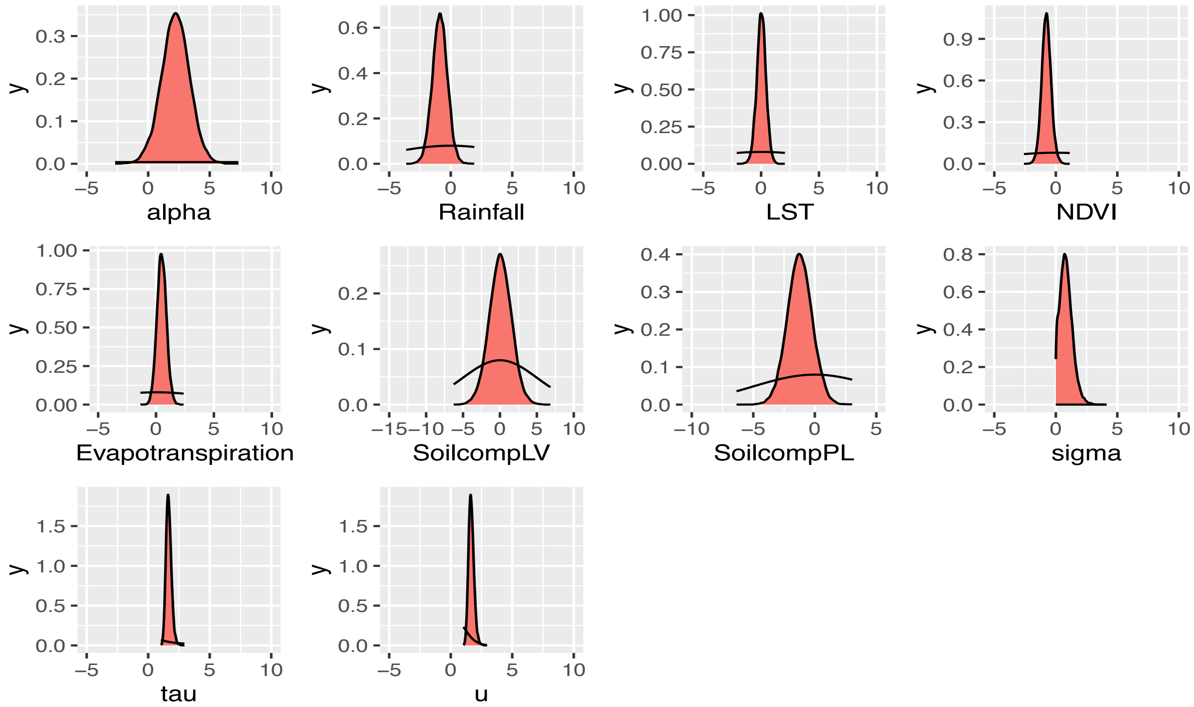

Figure S1: Prior against the posterior distribution. a) *Biomphalaria* sp. b) *Bulinus* spp.
