# Supplementary material for: A geospatial analysis of local intermediate snail host distributions provides insight into schistosomiasis risk within under-sampled areas of southern Lake Malawi
Source: Parasit Vectors. 2024 Jun 27;17:272. doi: 10.1186/s13071-024-06353-y (PMC11209974; doi:10.1186/s13071-024-06353-y)
Supplement: Supplementary file 9 — Additional file 9. Figure S1. [file 13071_2024_6353_MOESM9_ESM.pdf]

Additional file 9: Supplementary information

1D result

(a) *Biomphalaria* sp.

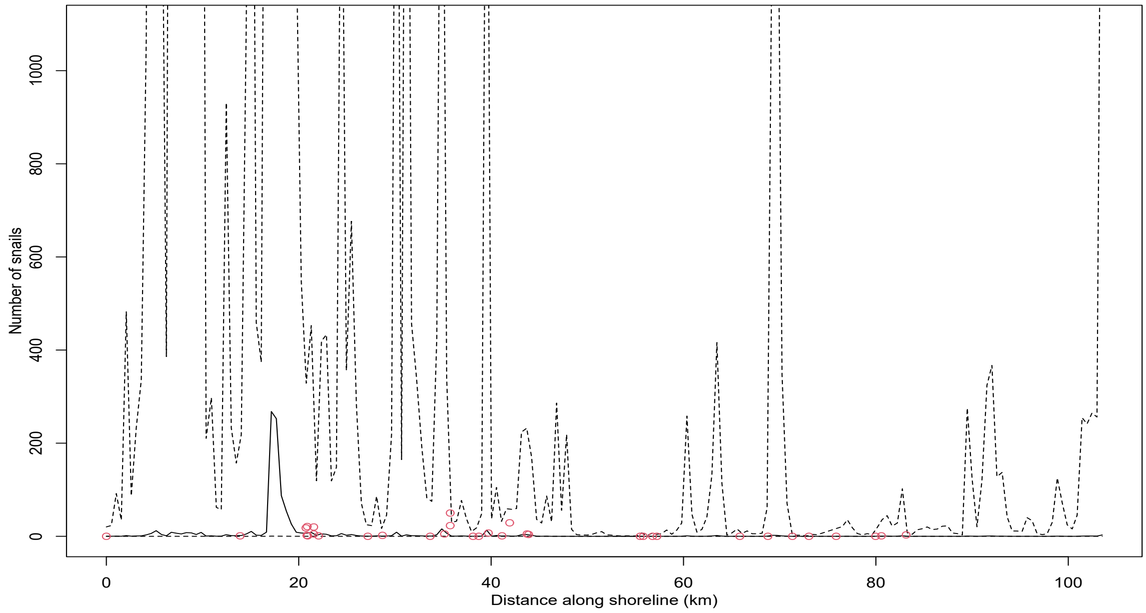

(b) *Bulinus* spp.

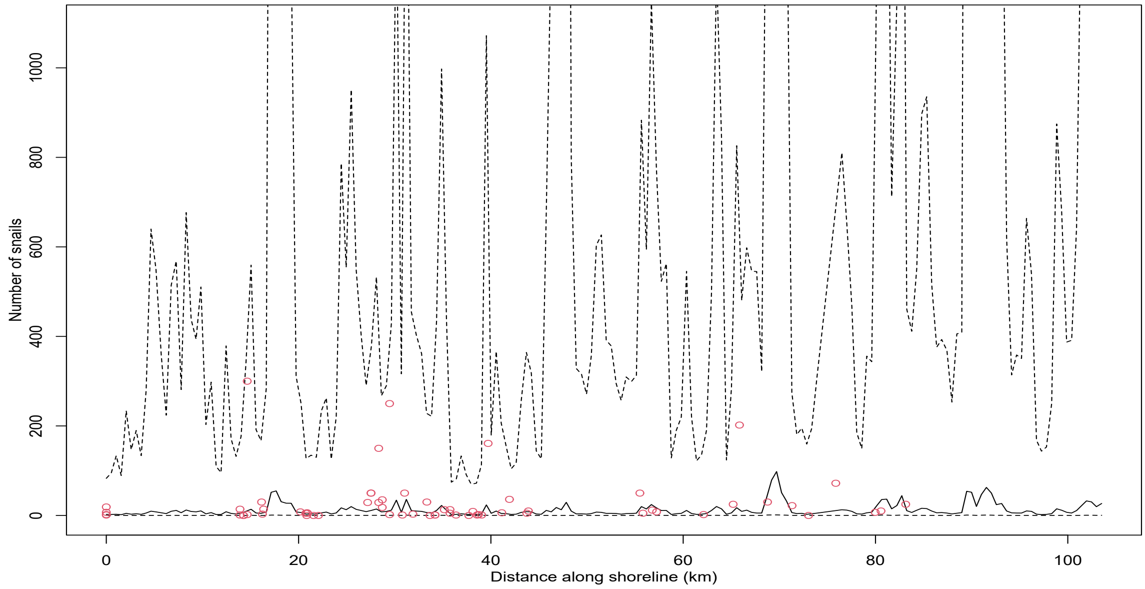

Figure S1: 1D medium Gaussian Prediction (GP) prediction (exponential covariance function) of number of snails log ($\widehat{\mu}_i$) against distance along the shoreline (km) a) *Biomphalaria* sp. b) *Bulinus* spp. Filled Straight line: Medium (50% credible intervals (Crl)) of GP prediction. Red circles: observed number of snails at sampling locations along the shoreline. Black faded lines: 95% Crl.
