# Supplementary material for: A geospatial analysis of local intermediate snail host distributions provides insight into schistosomiasis risk within under-sampled areas of southern Lake Malawi
Source: Parasit Vectors. 2024 Jun 27;17:272. doi: 10.1186/s13071-024-06353-y (PMC11209974; doi:10.1186/s13071-024-06353-y)
Supplement: Supplementary file 10 — Additional file 10. Figure S1, Figure S2 and Figure S3. [file 13071_2024_6353_MOESM10_ESM.pdf]

Additional file 10: Supplementary information

Bathymetric data

(a) *Biomphalaria* sp.

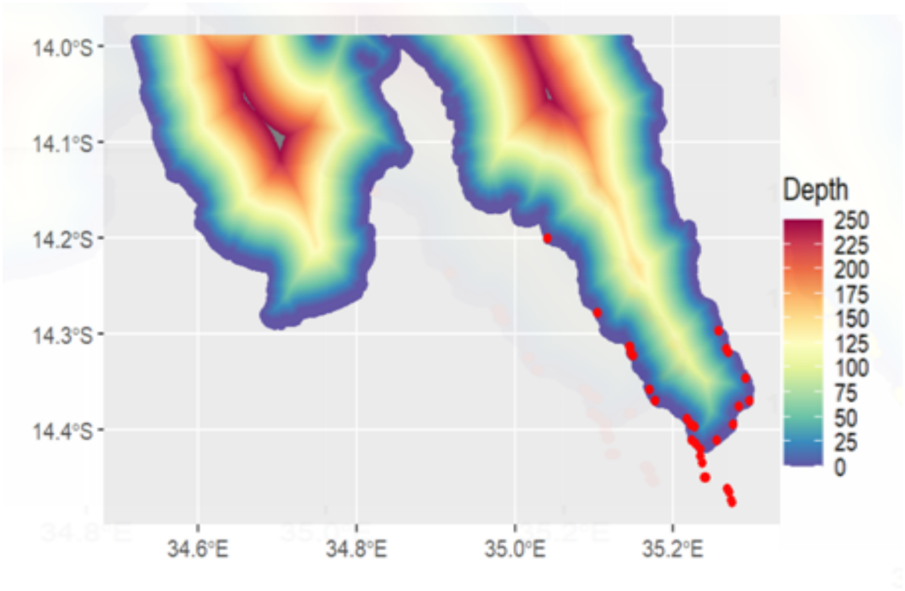

(b) *Bulinus* spp.

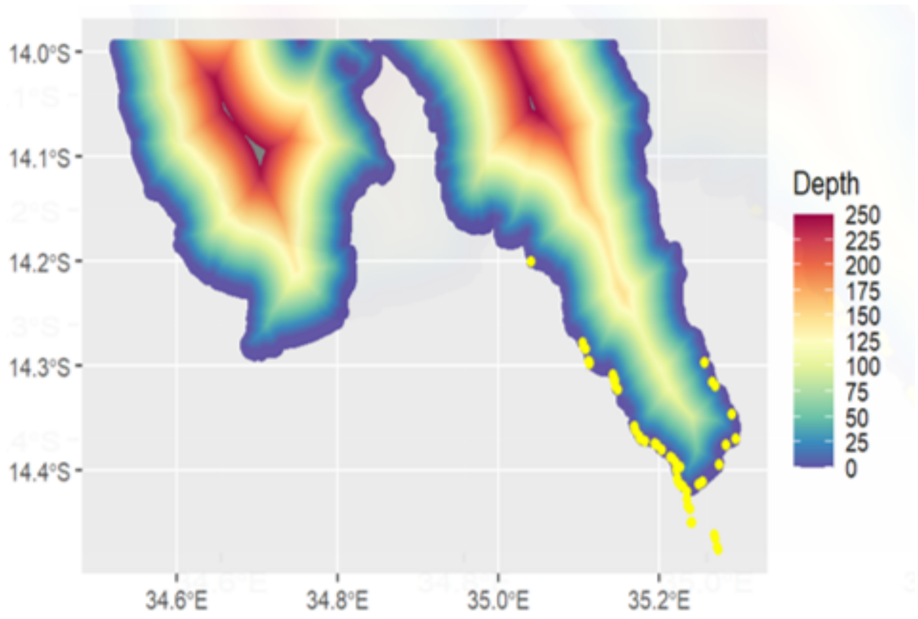

Figure S1: Bathymetric water depth (m) data for the shoreline with observed snails plotted a *Biomphalaria* sp. b) *Bulinus* spp..

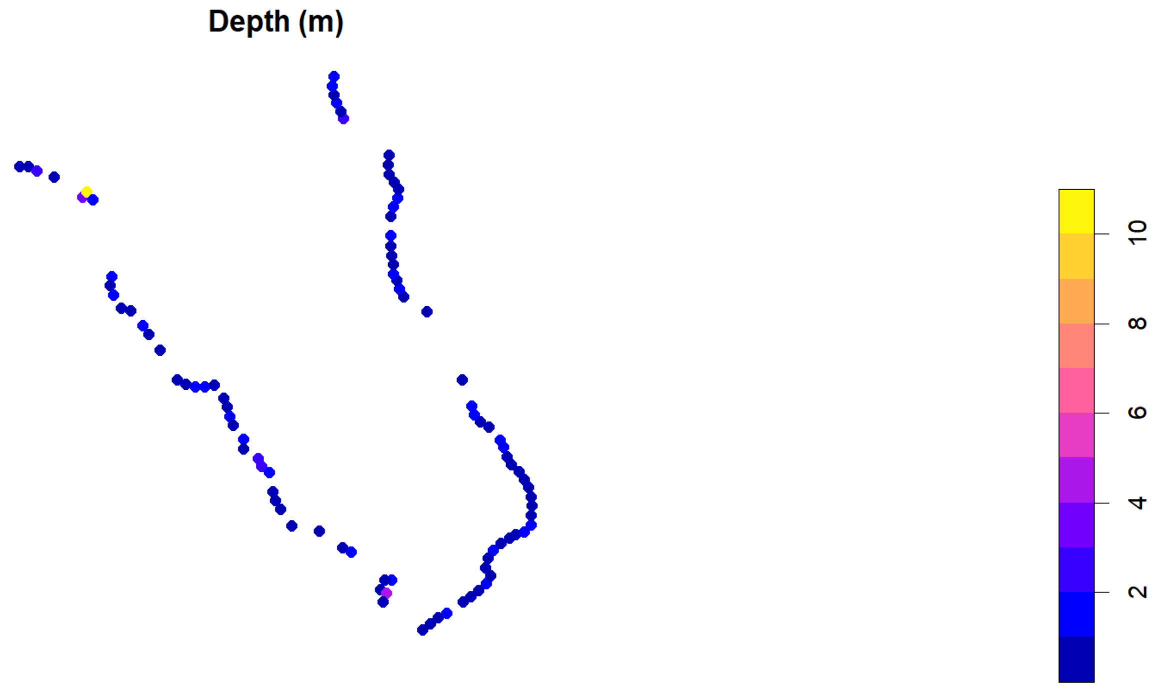

Figure S2: 2D plot of depth (m) extracted for the prediction points with 100km buffer.

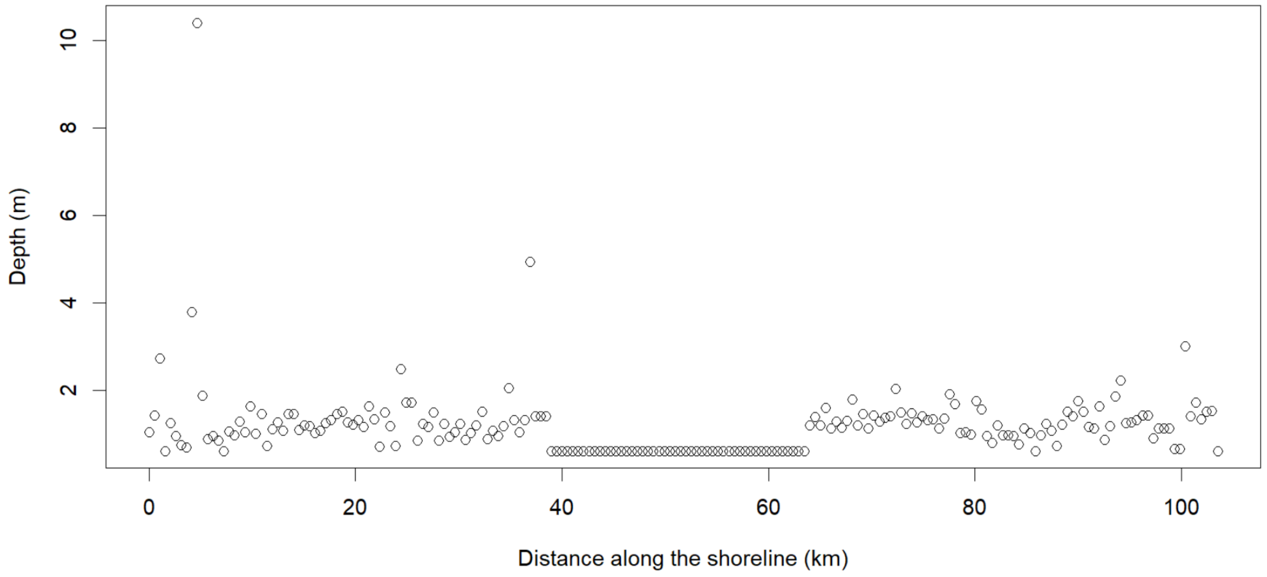

Figure S3: Depth (m) against distance along (km) the shoreline with 100km buffer and fill in NAs for the shoreline.
